# Core endophytic mycobiome in *Ulmus minor* and its relation to Dutch elm disease resistance

**DOI:** 10.3389/fpls.2023.1125942

**Published:** 2023-02-28

**Authors:** David Macaya-Sanz, Johanna Witzell, Carmen Collada, Luis Gil, Juan A. Martín

**Affiliations:** ^1^ Departamento de Ecología y Genética Forestal, Instituto de Ciencias Forestales (ICIFOR-INIA), CSIC, Madrid, Spain; ^2^ Department of Forestry and Wood Technology, Linnaeus University, Växjö, Sweden; ^3^ Departamento de Sistemas y Recursos Naturales, Escuela Técnica Superior de Ingeniería (ETSI) Montes, Forestal y del Medio Natural, Universidad Politécnica de Madrid, Madrid, Spain

**Keywords:** fungal endophytes, metabarcoding, plant-fungal interactions, Dutch elm disease, core microbiome, tree microbiome

## Abstract

The core microbiota of plants exerts key effects on plant performance and resilience to stress. The aim of this study was to identify the core endophytic mycobiome in *U. minor* stems and disentangle associations between its composition and the resistance to Dutch elm disease (DED). We also defined its spatial variation within the tree and among distant tree populations. Stem samples were taken i) from different heights of the crown of a 168-year-old elm tree, ii) from adult elm trees growing in a common garden and representing a gradient of resistance to DED, and iii) from trees growing in two distant natural populations, one of them with varying degrees of vitality. Endophyte composition was profiled by high throughput sequencing of the first internal transcribed spacer region (ITS1) of the ribosomal DNA. Three families of yeasts (Buckleyzymaceae, Trichomeriaceae and Bulleraceae) were associated to DED-resistant hosts. A small proportion (10%) of endophytic OTUs was almost ubiquitous throughout the crown while tree colonization by most fungal taxa followed stochastic patterns. A clear distinction in endophyte composition was found between geographical locations. By combining all surveys, we found evidence of a *U. minor* core mycobiome, pervasive within the tree and ubiquitous across locations, genotypes and health status.

## 1 Introduction

The endophytic assembly in deciduous plant tissues (e.g. annual plants, and deciduous leaves) is largely configured each season through horizontal transmission, when priority effects appear to be crucial (Toju et al., 2018b; Ridout et al., 2019; Debray et al., 2022). However, the assembly of endophytes in perennial organs (e.g. tree stems) is likely more complex (Saikkonen, 2007). Studies in crop plants and forest trees have reported consistent co-occurrence of endophytic assemblages known as core microbiomes, i.e., assemblages of microbes that constantly reside in the plant and are shared among conspecific hosts (Shade and Handelsman, 2012; Thomas et al., 2019; Noble et al., 2020). These core microbes are part of functional networks that positively or negatively affect host performance (Bonito et al., 2019). However, little is understood about core microbes of perennial organs and the extent to which their assembly is shaped by random colonization, environmental cues or active host recruiting factors (Müller et al., 2016). Perhaps because sampling in large tree crowns presents methodological difficulties, the diversity and spatial distribution of endophytes in long-lived trees remain largely unexplored.

Numerous environmental factors can potentially affect plant colonization by endophytes, including age, light availability, spatial distance from soil, and microclimate within the crown (Johnson and Whitney, 1989; Helander et al., 1993; Bahram et al., 2022). The endophytic composition can be also affected by host geographical location and host vitality (Agostinelli et al., 2018). Indeed, some endophytes that colonize long-lived trees are facultative saprotrophs or necrotrophs living in a cryptic phase (Carroll, 1988; Baum et al., 2003). Through environmental filtering, local climatic conditions (e.g. temperature, humidity and rainfall) can strongly influence the production and release of microbial propagules with potential to invade tree tissues (Zimmerman and Vitousek, 2012; Giauque et al., 2019). Furthermore, host-specific traits can drive an active recruitment of microbes (Cregger et al., 2018; Gallart et al., 2018). For instance, a genotype-dependent production of defense compounds against pathogens was shown to alter endophyte community assembly in maize (Saunders and Kohn, 2009). As a consequence of host and environmental effects on microbiomes, the composition of the surrounding vegetation and changes in land use can alter endophyte community at stand level (Li et al., 2019). In sum, endophyte assembly is conditioned by complex interactions among plants, microbes and the environment.

The current pandemic of Dutch elm disease (DED) is caused by *Ophiostoma novo-ulmi*. Since the beginning of the past century, DED has caused massive loss of elm trees native to Europe and North America (Martín et al., 2019b). The disease is vectored by elm bark beetles in the genera *Scolytus* and *Hylurgopinus*, or transmitted through root contacts. After inoculation, the fungus establishes in internal plant tissues, where it sporulates and spreads systemically, causing massive occlusion and embolism of xylem vessels. In most cases, infection ultimately leads to a wilt syndrome and tree death (Ouellette and Rioux, 1992), although some individuals are able to survive as recruiting trees through disease-resprouting cycles (Brasier and Webber, 2019). The composition of endophytic fungi in elms remains largely unexplored. A previous study showed that endophyte diversity in elms was influenced by host location and genotype (Martín et al., 2013), and that the diversity of the mycobiome in the xylem (but not in leaves or bark) of elm trees susceptible to DED was higher than in resistant trees. However, this study addressed only the culturable fraction of endophytes, which account for less than 5% of the total fungal richness within a tree (authors, personal observation).

Elm resistance to DED is affected by multiple factors, including the genetic make-up of hosts and pathogens, and their interaction with the environment (Martín et al., 2021). The role of microbiome in tree resistance remains poorly understood, although in ash dieback complex associations between endophytes and host genotypes seem to condition the outcome of disease (Griffiths et al., 2020). It is becoming clearer that certain endophytic infections trigger systemic responses in plants (Mejía et al., 2014) in certain cases priming plant defense against pathogens, as was recently evidenced in the case of the elm-*O. novo-ulmi* pathosystem (Martínez-Arias et al., 2021a). Some endophytes may also produce antimicrobial metabolites, enzymes, hormones and other bioactive compounds, enhancing host resistance (Hardoim et al., 2015; Busby et al., 2016; Martínez-Arias et al., 2021c). In particular, the core microbiome of a plant seems to exert key effects on plant performance and resistance to various stressors (Shade and Handelsman, 2012; Toju et al., 2018a). Following this concept, core taxa associated with elms probably perform essential functions, including protection against disease.

The general aim of this study was to identify the core endophytic mycobiome in *U. minor* stems as a first step to unravelling the ecology of elm microbial consortia. To address this aim we studied: i) the spatial variation of endophyte composition within the aerial part of a mature tree and between distant geographical locations; ii) the endophyte composition of ten *U. minor* trees showing a gradient of resistance level to *O. novo-ulmi*; and iii) the fungal composition of six large *U. minor* trees showing different vitality levels but growing in the same location.

## 2 Materials and methods

### 2.1 Plant material

To determine how tree stem fungal microbiome is structured, we sampled wood tissue from twigs (1-2 cm diameter) and trunks (5-cm cores at breast height) from trees at four locations in Spain in the spring of 2012. We focused on stem endobiome because it is a perennial tissue, in which microbiome interactions have time to evolve and mature, and because the agent responsible for DED is a vascular pathogen and therefore mostly interacts with the xylem microbiome. To prevent inclusion of epiphytic flora, the external layer of the bark (periderm) was manually extirpated after the collection. The stem tissues analyzed were xylem and the remaining phloem.

#### 2.1.1 Within-tree mycobiome variation

**Figure 1:**
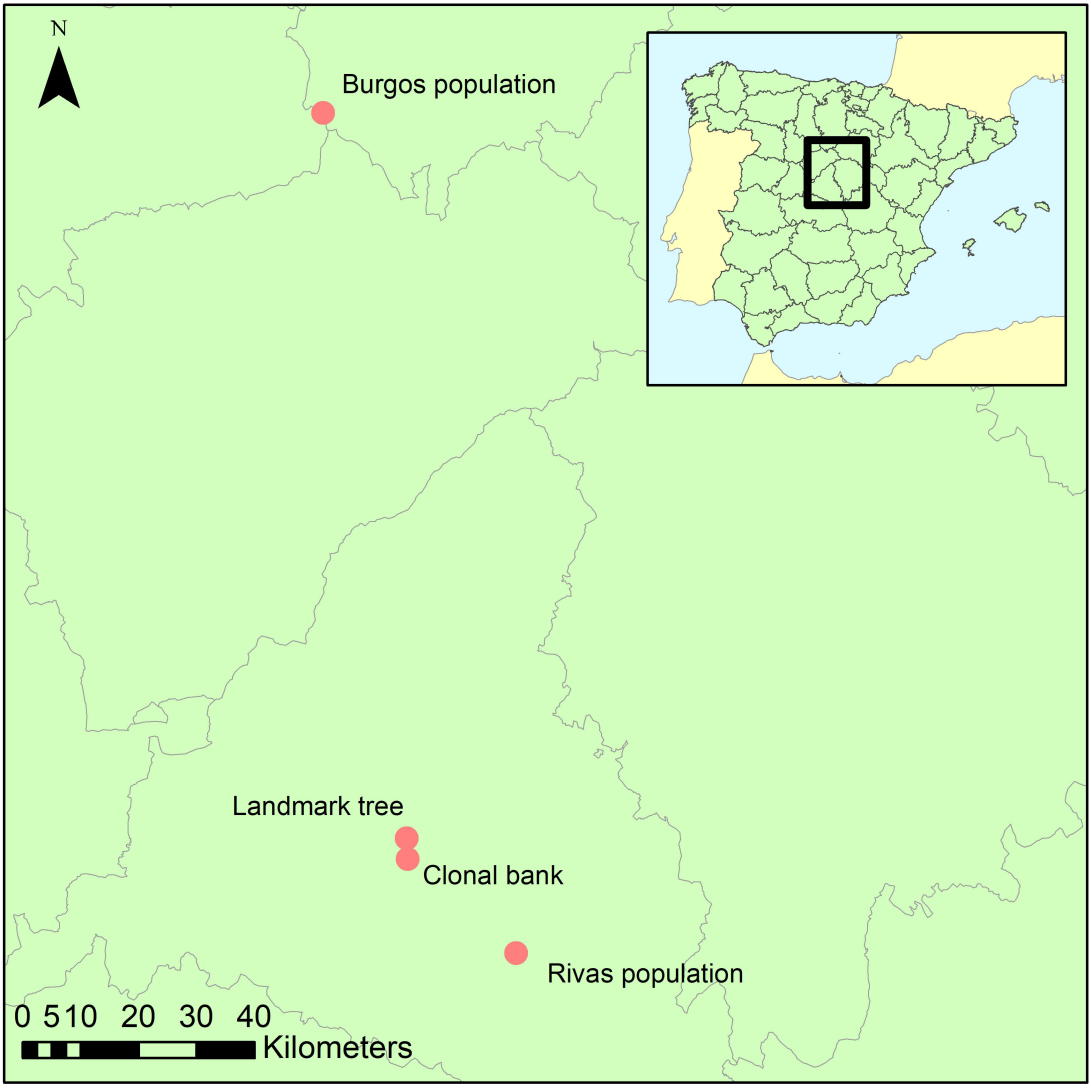
Geographical location of the four collection sites.

Ten spots were sampled within the crown and on the stem of a landmark *Ulmus minor* tree (Somontes, Madrid, Spain; **Figure 1**; ‘landmark tree’). The samples comprised eight twigs from the crown at four heights (3, 8, 13 and 18 m) and two orientations (north and south), and two trunk cores (same orientations). Cores were extracted using a sterilized core drill. The 25-m tree was a lingering monumental elm. Common garden tests on clones generated from its cuttings showed that the tree was not genetically resistant to DED (data not shown), and in 2014 it died after an exceptionally harmful DED outbreak.

#### 2.1.2 Wood mycobiome and elm DED resistance

The second sampling was at the elm clonal bank (common garden setup) at the Puerta de Hierro Forest Breeding Centre (Madrid; **Figure 1**), the headquarters of the Spanish elm breeding program. The clonal bank has around 250 genotypes from Spain, including seven DED-resistant genotypes (Martín et al., 2015). Four twigs were collected from scaffold branches in 10 trees (**Supplementary Table 1**) catalogued as resistant (n=3; V-AD2; M-RT1.5; M-DV5), intermediately susceptible (n=4; CR-RD2; GR-HL2; J-CA2; MA-PD2), or susceptible (n=3; GR-DF3; M-DV1; TO-PB1). Samples were collected at four spots per tree to ensure accurate representation of the endophyte composition and mitigate any effect of local infections (see below). All twigs were collected from the lower half of the crown, to a height of 4 m.

The level of resistance to DED of the 10 *U. minor* clones sampled at the clonal bank was determined during screening tests at the Spanish elm breeding program at Puerta de Hierro Forest Breeding Centre (Madrid, Spain) (**Supplementary Table 1**, **Supplementary Text**). The 10 trees sampled have been never artificially inoculated with the DED pathogen.

#### 2.1.3 Variation in trees differing in vitality phenotype

Following the same protocol as in the clonal bank, twigs from six trees were collected from a natural *U. minor* stand in the municipality of Rivas-Vaciamadrid (Rivas population; ‘Madrid province’; **Figure 1**). This population lacks genetically resistant clones (tested in a common garden) but has not been eradicated by DED. The reasons behind this elusion are unclear but could be due to phenotypic avoidance due to the effect of biotic or abiotic factors. The stand is nonetheless showing clear signs of dieback, in part because of DED infections but various other undetermined causes might be playing a role. Most trees in this stand belong to the susceptible *U. minor* var. *vulgaris*. This taxon presents very low genetic variability, because it originated from a single *U. minor* tree, the Atinian elm (Gil et al., 2004). Indeed, these trees are genetically similar to the clone TO-PB1, another *U. minor* var. *vulgaris* specimen held at the Breeding Centre (and included in the clonal bank collection). We collected samples from trees ranging various health statuses (**Supplementary Figure 1**). Those health statuses (named RIV1 to RIV6) were scored visually from 1 (no symptoms) to 6 (profuse dieback symptoms).

#### 2.1.4 Variation among geographical locations

Using the same protocol as in the clonal bank, three trees from a small, natural stand in the province of Burgos (approximately 150 km north of the other locations; **Figure 1**) were sampled to provide a background reference of endophyte diversity and composition of the populations in Madrid province.

### 2.2 DNA isolation, amplification and NGS

After the collection, samples were sterilized, peeled, frozen and ground. All these steps were carried out in a laminar flow cabinet to minimize contaminations. The four twig samples taken from each individual tree at the clonal bank, Rivas and Burgos populations were combined and milled together, resulting in one pool of wood powder per sampled tree. DNA was isolated from the powder after enzymatic digestion to improve recovery of fungal DNA. Zirconium oxide beads were added during vortexing to increase cell wall lysis. Endophyte composition was profiled by high throughput sequencing of the first internal transcribed spacer region (ITS1) of the ribosomal DNA. Sequencing effort was uneven among experiments, prioritizing the landmark tree samples, which were also the first to be processed to determine the level of resolution needed in subsequent experiments. The clonal bank experiment followed in sequencing effort, to attain accurate values of endophyte abundance for identifying potential associations with DED resistance. The Burgos population was only shallowly sequenced since, as an outgroup, was only intended to test for ubiquity of microbiome elements detected in the other populations. DNA amplification was performed in two steps: (1) to cover the target region with oligonucleotides that contained the specific fungal primer ITS1-F (Gardes and Bruns, 1993) or the non-specific primer ITS2 (White et al., 1990); (2) to attach the adaptors for the sequencing platform. After the second PCR, the product of all the samples was quantified, pooled equimolarly and pyrosequenced in a 454 GS FLX Titanium platform (Roche, Basel, Switzerland). A negative control sample was created by autoclaving collected twigs three times and then applying to them the same protocols previously described. A more detailed description of these methods is available in the Supplementary Text.

### 2.3 Bioinformatic pipeline

The bioinformatic treatment of pyrosequencing output was performed following the guidelines of Lindahl et al. (2013). Demultiplexing, denoising, dereplication, dechimerization and sequence truncation processes were carried out using the default values of the RunTitanium script developed in AmpliconNoise v1.29 (Quince et al., 2011; **Supplementary Text**). The ITS1 region was then extracted from the sequences using FungalITSextractor (Nilsson et al., 2010).

Although AmpliconNoise creates OTUs (Operational Taxonomic Units) by collapsing identical sequences, we further clustered them with the grammar-based software GramCluster 1.3 (Russell et al., 2010) in greedy mode to build new OTUs, allowing higher variation among sequences. This program was run on the whole dataset (i.e. pooling the output of all samples) to build OTUs across all samples, allowing subsequent among-sample comparisons.

### 2.4 Taxonomic assignment

Taxonomic composition was investigated using the naïve Bayesian classifier method implemented in R package dada2 v. 1.22.0 (Wang et al., 2007; Callahan et al., 2016). We used the last available UNITE release (16/10/2022) (Kõljalg et al., 2005; Nilsson et al., 2019; Kõljalg et al., 2020) as the reference curated database. For OTUs of special interest, we carried out BLAST searches on the NCBI database to double-check the assignment provided by dada2 using the UNITE database.

### 2.5 Diversity estimates and hypothesis contrasts

Commonly used diversity indices were estimated for each sample collected, using the counts per OTU as taxonomic information. Shannon’s *H* and Simpson’s *λ* indices, and species richness on counts rarefacted to 500, were calculated using R package “vegan” v. 2.6.4 (Oksanen et al., 2015). Statistical analyses were performed taking into account that count data in these types of studies follow a negative binomial distribution as in RNA-seq experiments (McMurdie and Holmes, 2014). As suggested by these authors, R package DESeq2 v. 1.34.0 (Love et al., 2014), which is designed to construct negative binomial models, was used to examine the data and test for associations between taxonomic group abundance and resistance to DED. In order to explore the structure of the samples, DESeq2 was used to perform a variance-stabilizing transformation of the OTU counts to conduct a standard Principal Components Analysis. Tests for associations were run on the clonal bank samples, setting crown wilting percentage (as a proxy of resistance) as the only explanatory variable. Significance was calculated with a Wald test and adjusted for multi-testing using the default DESeq2 approach that estimates False Discovery Rate adjusted P-values (more details in Supplementary Text). Given the unreliable taxonomic certainty of OTU formation through clustering and the possible redundancy in ecological function of closely related species and genera, we decided to focus on the higher taxonomic levels (such as family and order).

### 2.6 Core microbiome demarcation

The distributions of number of samples in which each OTU was present (OTU incidence distribution) were used to determine which OTUs were putatively from the core microbiome, following the concept of Shade and Handelsman (2012). The expected pattern of incidence of OTUs, if their occurrence probability is low and mostly based on randomness (i.e. local infections rather than core microbiome), must agree with a Poisson or negative binomial distribution. Therefore, if the OTU incidence distribution departs from that hypothesized behaviour, it can be assumed that non-local infections are occurring. Consequently, we selected more than seven samples as the threshold value in both the landmark tree and the clonal bank because it was where the distributions clearly diverged from Poisson distributions (see Results). Thus, OTUs present in more than seven spots of the landmark tree or in more than seven trees of the clonal bank, and also present in at least two out of the four locations, were considered core members.

## 3 Results

### 3.1 Sampling effort and saturation

**Figure 2:**
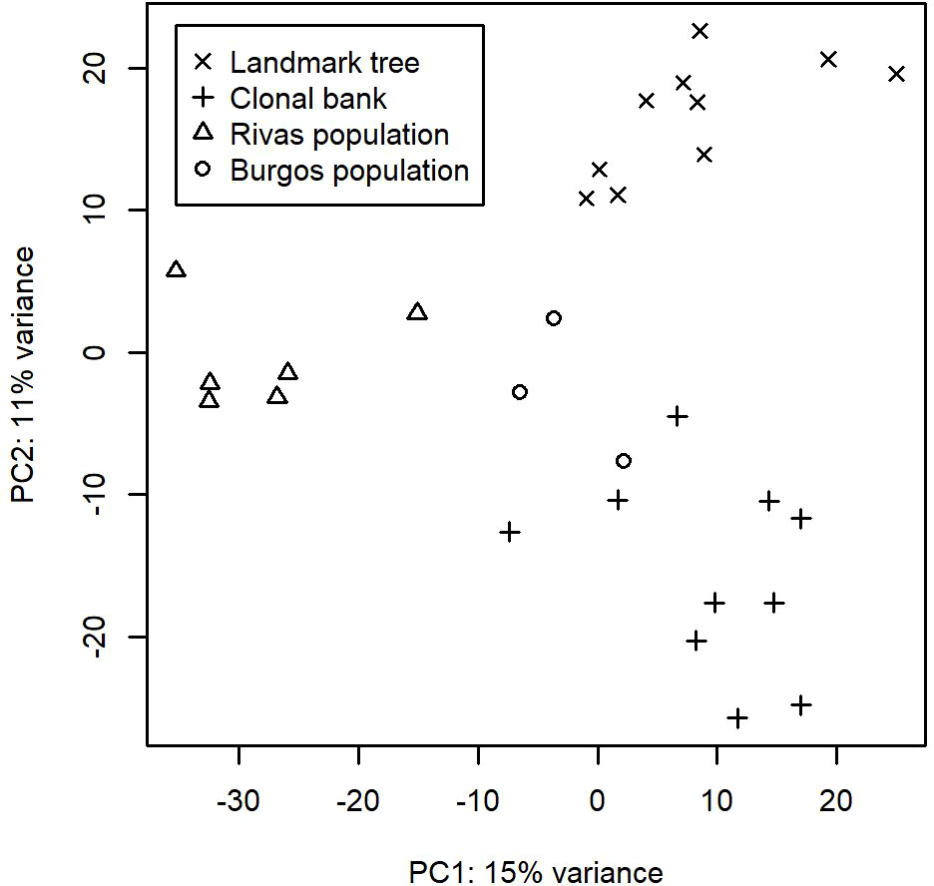
First two axes from the Principal Component Analyses performed on the OTU counts after variance stabilizing transformation.

After running the bioinformatic pipeline, we obtained 106,047 informative reads (considered counts). These were grouped by GramCluster into 435 clusters (considered OTUs henceforth). Out of these, 74 were singletons, 40 doubletons and 23 tripletons. A further 263 OTUs were represented by more than five reads. Five OTUs belonged to kingdoms other than fungi. Those OTUs plus the ones represented by singletons or doubletons were discarded for further analyses. To ensure a more accurate OTU richness comparison, we rarefied the count data to 500 reads per sample. The mean values (± s.e.) of rarefied OTUs ranged from 64.4 ± 3.3 in one of the lower resprouted branches of the Somontes tree to 15.6 ± 2.1 in one sample from the Rivas stand (RIV2, with advanced dieback). Rarefaction curves supported the figures observed by the rarefaction to 500 reads and indicated that the sampling effort was sufficient to capture the richness trends of each sample (**Supplementary Figures 2**, **3**). Principal Component Analysis showed a separation between sites (**Figure 2**).

Across the total sample set, 103 families, 48 orders, 17 classes and 3 phyla were detected. Out of the 317 OTUs not discarded, 293 were assigned to a phylum, 267 to a class, 256 to an order and 228 to a family. Genus was provided for 203 OTUs, and species for 131. However, both genus and species assignments cannot be considered reliable due to the reduced taxonomic resolution of the ITS1.

### 3.2 Within-tree distribution of endophytes

**Figure 3:**
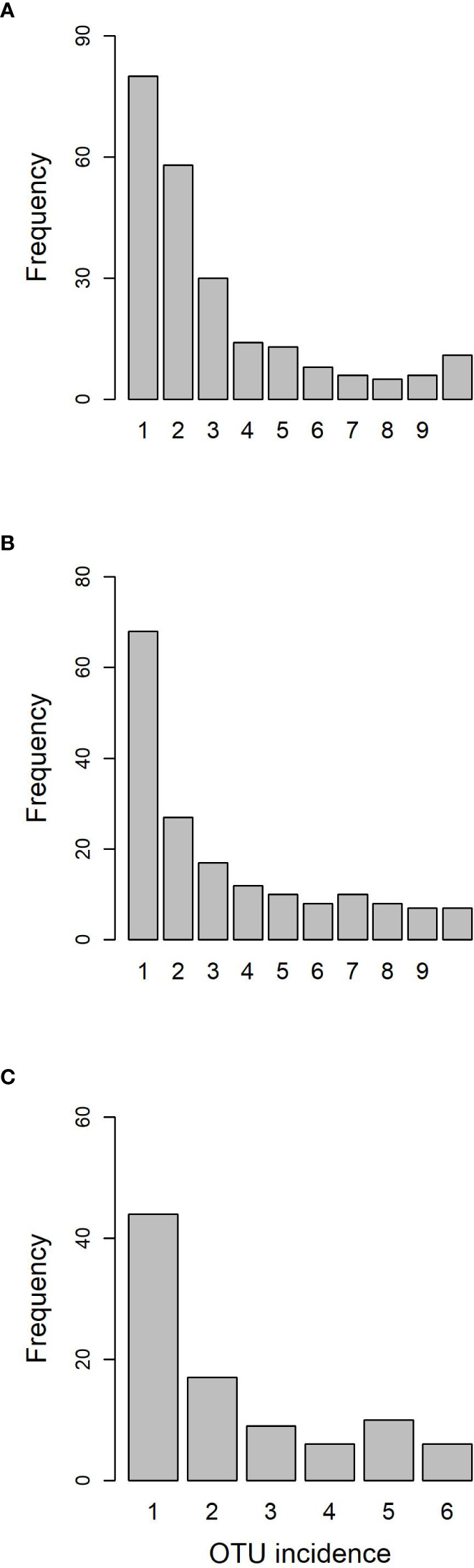
OTU frequency spectra for **(A)** landmark tree, **(B)** clonal bank and **(C)** Rivas population.

**Figure 4:**
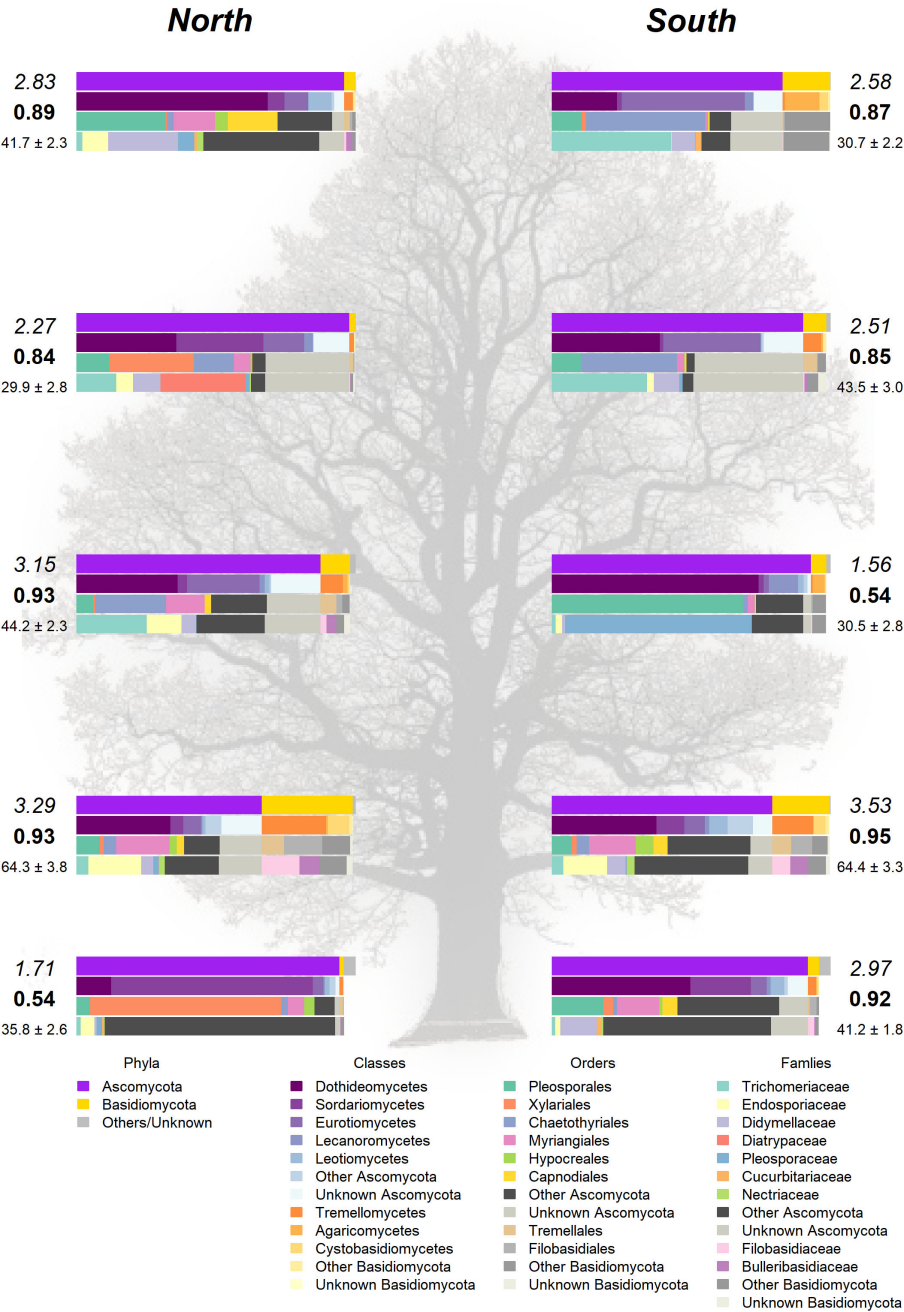
Taxonomic composition in the landmark tree. Only the most relevant taxa are shown. Colored bars represent the frequency of taxa at the levels of phylum, class, order and family (top to bottom). Numbers next to the bars indicate the Shannon (italics) and Simpson (bold) indices and the OTU richness rarefacted to 500 reads (with standard error). (Background image source: Tree Silhouette copy by Bob G in flickr, licensed under CC BY-NC-SA 2.0).

The Somontes tree had 68,612 reads passing filtering, clustered into 231 OTUs (8 singletons, 2 doubletons and 14 tripletons, just considering the landmark tree counts). Regarding incidence, 11 OTUs were present in all the in-tree spots sampled and 22 were present in at least eight (**Table 1**, **Figure 3A**). A further 80 OTUs were present in just one spot and 58 were present in two (**Figure 3A**). The number of OTUs at higher abundance in the tree did not follow a purely rare event distribution such as the Poisson or negative binomial distribution, as seen in the smooth but distinguishable peak at the end of the distribution (**Figure 3A**). Three phyla, 15 classes, 41 orders and 81 families were detected within the tree (**Figure 4**). Across the tree, the levels of diversity (measured as Shannon’s *H*, Simpson’s *λ* and rarefied OTU richness) were generally high, with the following deviations: (i) the two lowest branches, produced from resprouts from the trunk, displayed remarkably higher levels of diversity; (ii) one sample from the trunk and one from the middle crown exhibited low values of both *H* and *λ*.

**Table 1:** OTUs present in at least eight samples of the landmark tree or the clonal bank.

*OTU id*	*Phylum*	*Order*	*Class*	*Family*	*Genus*	*NL*	*NC*	*NT*	*Npop*
OTU_0	Ascomycota	Dothideomycetes	Myriangiales	NA	NA	7	**9**	21	4
OTU_2	Ascomycota	Dothideomycetes	Pleosporales	Pleosporaceae	*Alternaria*	**8**	7	23	4
OTU_6	Ascomycota	Dothideomycetes	Dothideales	Saccotheciaceae	*Aureobasidium*	7	**10**	26	4
OTU_7	Ascomycota	Dothideomycetes	Myriangiales	Endosporiaceae	*Endosporium*	**10**	**10**	26	4
OTU_8	Ascomycota	Dothideomycetes	Pleosporales	Cucurbitariaceae	NA*	2	**9**	19	4
OTU_10	Ascomycota	Dothideomycetes	Pleosporales	Didymellaceae	NA	**10**	**10**	29	4
OTU_13	Ascomycota	Orbiliomycetes	Orbiliales	Orbiliaceae	*Retiarius*	6	**8**	15	3
OTU_14	Ascomycota	Dothideomycetes	Mycosphaerellales	Teratosphaeriaceae	*Lapidomyces*	3	**8**	11	2
OTU_15	Basidiomycota	Tremellomycetes	Filobasidiales	Filobasidiaceae	*Filobasidium*	**10**	**9**	25	4
OTU_16	Ascomycota	Dothideomycetes	Mycosphaerellales	Extremaceae	*Petrophila*	4	**8**	13	3
OTU_18	Ascomycota	Sordariomycetes	Hypocreales	Incertae sedis	*Trichothecium*	0	**10**	16	3
OTU_21	Ascomycota	NA	NA	NA	NA	**8**	**8**	19	4
OTU_23	Ascomycota	NA	NA	NA	NA	**10**	7	17	2
OTU_24	Ascomycota	Leotiomycetes	Thelebolales	Pseudeurotiaceae	NA*	**10**	**9**	23	4
OTU_25	Ascomycota	Eurotiomycetes	Eurotiales	Aspergillaceae	*Penicillium*	**10**	5	17	4
OTU_27	Ascomycota	Lecanoromycetes	Caliciales	Physciaceae	*Rinodina*	**10**	**10**	21	3
OTU_29	Ascomycota	Dothideomycetes	NA	NA	NA	**9**	**8**	20	3
OTU_32	Ascomycota	Dothideomycetes	Mycosphaerellales	NA	NA	2	**8**	11	3
OTU_33	Ascomycota	Dothideomycetes	Capnodiales	Cladosporiaceae	*Cladosporium*	**10**	**10**	28	4
OTU_34	Ascomycota	Sordariomycetes	Xylariales	Leptosilliaceae*	Leptosillia*	**8**	3	13	3
OTU_35	Ascomycota	Dothideomycetes	Botryosphaeriales	Botryosphaeriaceae	*Neofusicoccum*	**9**	7	16	2
OTU_38	Ascomycota	Sordariomycetes	Xylariales	Xylariaceae	Entoleuca*	0	**9**	15	3
OTU_40	Ascomycota	Eurotiomycetes	Chaetothyriales	Trichomeriaceae	NA	**9**	**9**	18	2
OTU_41	Ascomycota	Eurotiomycetes	Chaetothyriales	Trichomeriaceae	*Knufia*	**10**	**10**	29	4
OTU_46	Ascomycota	NA	NA	NA	NA	**10**	**9**	26	4
OTU_51	Ascomycota	NA*	NA*	NA*	NA*	6	**8**	17	4
OTU_65	Basidiomycota	Tremellomycetes	Tremellales	Bulleribasidiaceae	*Vishniacozyma*	**9**	6	23	4
OTU_66	Ascomycota	Dothideomycetes	Pleosporales	Didymellaceae	NA	**10**	7	25	4
OTU_71	Basidiomycota	Cystobasidiomycetes	Incertae sedis	Buckleyzymaceae	*Buckleyzyma*	**9**	**8**	22	4
OTU_80	Ascomycota	Eurotiomycetes	Chaetothyriales	NA*	NA*	**9**	5	22	4
OTU_102	Ascomycota	Dothideomycetes	Pleosporales	Pleosporaceae	*Alternaria*	**8**	2	11	3
OTU_178	Ascomycota	NA	NA	NA	NA	**8**	1	9	2

Taxonomic assignment is based on ITS1 DNA similarity with UNITE database. Star (*) indicates assignment change after check in the NCBI database. The final columns show the number of samples in the landmark tree (*NL*), the clonal bank (*NC*) and the total sample set (*NT*) and the number of geographical locations (*Npop*) where the OTUs were detected. Bold numbers indicate presence in eight or more collected samples. NA indicates Not Assigned

### 3.3 Endophyte diversity in relation to DED resistance

**Figure 5:**
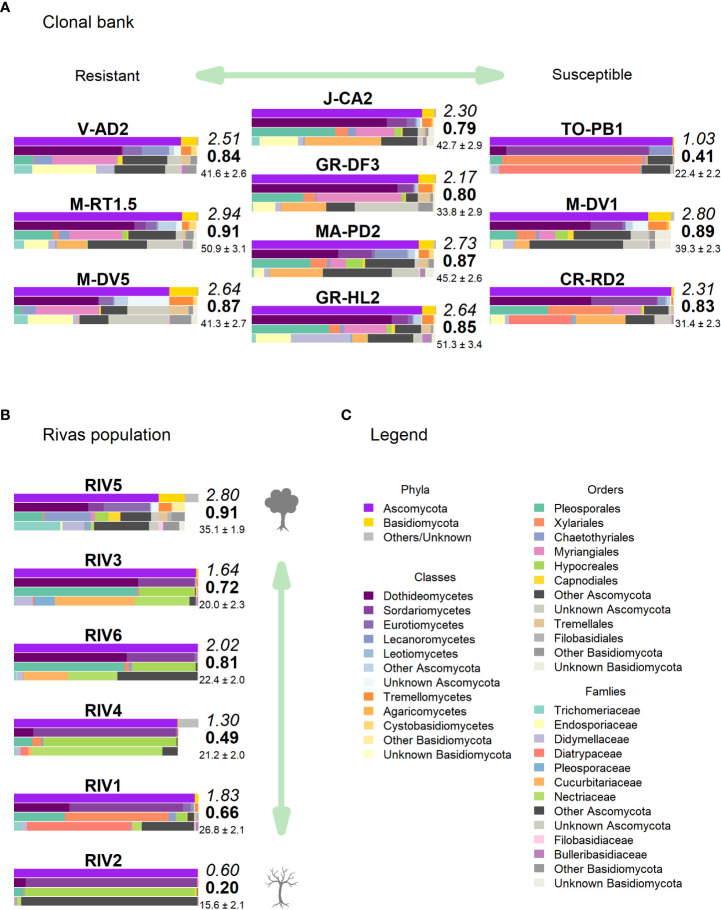
Taxonomic composition in **(A)** clonal bank and **(B)** Rivas population. Only the most relevant taxa are shown. Colored bars represent the frequency of taxa at the levels of phylum, class, order and family (top to bottom), following legend color code **(C)**. Numbers next to the bars indicate the Shannon (italics) and Simpson (bold) indices and the OTU richness rarefacted to 500 reads (with standard error). (Tree icon sources: minimal tree simple SVG Silh, licensed under CC0 1.0 and tree-304418 by Clker-Free-Vector-Images in pixabay under Pixabay licence).

High-throughput sequencing on the 10 trees of varying levels of resistance to DED from the clonal bank at Puerta de Hierro breeding center produced 20,534 sequences after filtering. The sequences were clustered into 173 OTUs: 20 singletons, 11 doubletons and 19 tripletons. Similar to the results in the Somontes tree, most OTUs were present in just one sample (67), two samples (27) or three samples (17). However, the counts did not drop at a rate consistent with a Poisson process, and reached a stable level beyond five samples (**Figure 3B**). In total, two phyla, 15 classes, 34 orders and 68 families were detected (**Figures 5A, C**).

Clone TO-PB1 (susceptible) displayed the lowest levels of diversity (*H* = 1.03). Conversely, the resistant clone M-RT1.5 showed the highest overall diversity estimates (*H* = 2.94). GR-HL2 (susceptible) and MA-PD2 (moderately resistant) also displayed high diversity values. Wilting after DED inoculation (used as a proxy of susceptibility) was not significantly correlated with any of the diversity estimates, indicating the absence of a strong correlation between diversity estimates and resistance to DED. However, the limited sample size (n = 10) may have prevented detection of a more subtle correlation.

**Figure 6:**
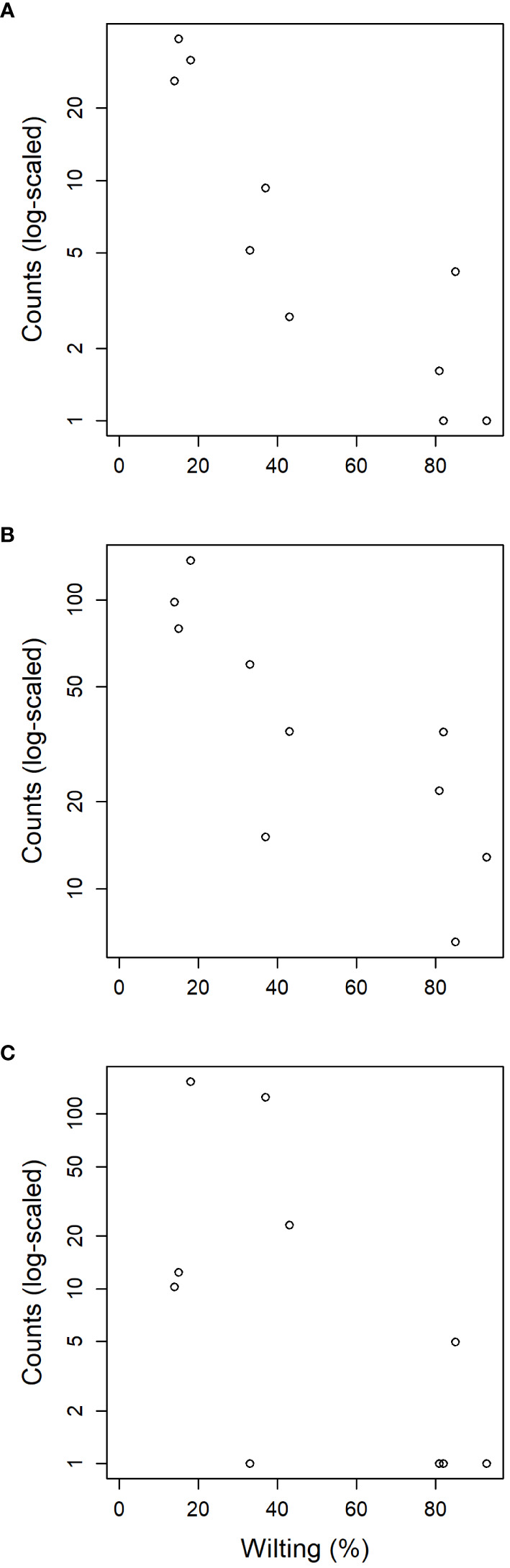
Relation between susceptibility to DED (measured as leaf wilting percentage) of the ten clonal bank genotypes and the normalized counts detected from reads of endophytic fungal families **(A)** Buckleyzymaceae, **(B)** Trichomeriaceae and **(C)** Bulleraceae.

The tests of association between wilting and taxa abundance produced unambiguous hits (**Table 2**). Three families and three orders were significantly associated with resistance and one family and order was associated with susceptibility. The family with the highest association was Buckleyzymaceae (**Figure 6A**), a Basidiomycota of the Cystobasidiomycetes class and undefined order (*Incertae sedis*). It had lower support at OTU level, represented by the genus *Buckleyzyma* (OTU_71). The next most significant hit was from the family Trichomeriaceae, Ascomycota (**Figure 6B**), a recently circumscribed family in the order Chaetothyriales, excised from family Herpotrichiellaceae. It was also supported, but to a lesser degree, by the hit at OTU level, in OTU_41 assigned to the genus *Knufia*. The next and least significant hit at family level was Bulleraceae (**Figure 6C**), echoing at order level as Tremellales (Basidiomycota). Two OTUs (OTU_70 and OTU_55) were significant and belonged to the genera *Genolevuria* (based on UNITE) or the related *Cryptococcus* (based on NCBI). All these taxa were negatively associated with susceptibility (proxied as wilting). Family Diatrypaceae was positively associated with susceptibility, and this result was reproduced with stronger support at order level (Xylariales) and at class level (Sordariomycetes). Also, OTU_1 and OTU_19 (Sordariomycetes) were positively associated to DED susceptibility, being the former assigned by dada2 to the genus *Anthostoma* and by BLAST into NCBI’s GenBank to *Lopadostoma* but both with suboptimal identity (< 95%, due to a 11-bp indel), and the latter assigned *via* dada2 only at order level (Hypocreales), but *via* BLAST into NCBI’s GenBank to *Annulohypoxylon multiforme*, Xylariales (>99% identity). These findings hint at a general relationship between the Sordariomycetes and susceptibility.

**Table 2:** Taxa with significant positive or negative associations (*padj* < 0.1; *p-value* < 0.05 for OTUs) with resistance to DED.

Taxon	*baseMean*	*log2FC*	*lfcSE*	*stat*	*p-value*	*padj*	*Family*	*Order*
Class								
Cystobasidiomycetes	24.343	-2.038	0.475	-4.292	0.00002	0.00027		
Sordariomycetes	762.750	2.178	0.538	4.051	0.00005	0.00038		
Eurotiomycetes	71.577	-0.979	0.327	-2.994	0.00275	0.01375		
Order								
Xylariales	676.281	2.719	0.560	4.852	0.00000	0.00004		
Cystobasidiomycetes *incertae sedis*	19.118	-1.896	0.488	-3.886	0.00010	0.00153		
Chaetothyriales	59.560	-0.881	0.365	-2.410	0.01595	0.15951		
Tremellales	64.275	-0.977	0.426	-2.294	0.02180	0.16351		
Family								
Buckleyzymaceae	11.154	-2.128	0.572	-3.723	0.00020	0.01102		
Diatrypaceae	784.043	5.423	1.557	3.484	0.00049	0.01385		
Trichomeriaceae	49.146	-1.170	0.362	-3.233	0.00123	0.02288		
Bulleraceae	32.251	-2.889	0.982	-2.943	0.00325	0.04551		
OTU								
OTU_1	762.255	5.413	1.552	3.488	0.00049	0.05298	Diatrypaceae	Xylariales
OTU_70	19.432	-3.625	1.171	-3.097	0.00196	0.09598	Bulleraceae	Tremellales
OTU_71	13.338	-2.251	0.751	-2.998	0.00272	0.09598	Buckleyzymaceae	*Incertae sedis*
OTU_19	4.591	2.510	0.878	2.860	0.00424	0.09598		Hypocreales
OTU_55	20.021	-3.811	1.338	-2.848	0.00440	0.09598	Bulleraceae	Tremellales
OTU_41	49.449	-1.475	0.567	-2.602	0.00928	0.16857	Trichomeriaceae	Chaetothyriales

The test of association was performed by a Wald test. Column *baseMean* shows the mean of normalized counts; *log2FC*: estimate of the effect size scaled to the log2 of fold change; *lfcSE*: standard error of this estimate; *stat*: value of the Wald test statistic; and *p-value* and *padj*: respectively, the raw and the adjusted (for multiple tests) probabilities that the observed statistic is part of the null distribution. These columns correspond to the output of the function DESeq from R package DESeq2. A positive fold change indicates association with susceptibility to DED.

### 3.4 Endophytic mycobiome in trees representing a gradient of vitality

The six samples collected in the natural riparian stand at Rivas-Vaciamadrid municipality from trees at varying stages of dieback produced 13,408 reads, clustered into 92 OTUs: 16 singletons and 11 doubletons. Forty-eight were represented by more than five reads. Only six OTUs were present in all trees and 10 were present in five samples (**Figure 3C**). The secondary peak found in the OTU incidence distribution was not in the total number of samples (n = 6) but in n = 5.

None of these OTUs was identified as genus *Ophiostoma* or order *Ophiostomatales*, even though the UNITE database included several accessions for both *O. ulmi* and *O. novo-ulmi*, and it was undoubtedly detected as singleton in two trees of the clonal bank (GR-DF3 and V-AD2). The most affected tree (RIV2) and two trees with moderate dieback (RIV1 and RIV4) were dominated by Sordariomycetes: RIV1 was rich in Diatrypaceae and RIV2 in Bionectriaceae (**Figures 5B, C**). Both RIV4 (moderate dieback) and RIV6 (incipient dieback) had Nectriaceae as the most abundant family, although it was also abundant in the healthy RIV3. The two healthy trees (RIV3 and RIV5) were more infected than the other trees by Dothideomycetes and Eurotiomycetes. For diversity, RIV5 exhibited the highest values in all three indices calculated (Shannon’s *H*, Simpson’s *λ* and rarefied OTU richness). The affected RIV1 and RIV6 displayed high values of *H* and richness, and RIV3 (healthy) and RIV6 had high values of *λ*. The tree with lowest vitality (RIV2) had the lowest diversity values.

The healthiest tree (RIV5) displayed a clearly distinct pattern that was much richer in Basidiomycota (**Figure 5B**). Trichomeriaceae was the most common family in this tree, followed by Saccotheciaceae. The microbiome of RIV2, a tree with low vitality, was dominated by Bionectriaceae (OTU_147, identified as genus *Geosmithia* both in UNITE and NCBI; 100% of identity). This OTU was virtually absent in the other samples, except in the healthiest (RIV5), where it was not abundant but had a significant presence.

Regarding the taxa significantly associated with DED resistance, Buckleyzymaceae (represented mostly by OTU_71) was virtually absent from the population. Trichomeriaceae (represented mostly by OTU_41) was present in all trees but was much more abundant in RIV1 (dieback) and RIV5 (very healthy). Bulleraceae was slightly present in the healthiest tree RIV5. The single OTU associated with increased DED susceptibility (OTU_1; Diatrypaceae) was very abundant in RIV1 (dieback).

### 3.5 Patterns across the four sites – core fungal endobiome of *U. minor*

To assess the extent of ubiquity of the most common OTUs, we examined the patterns of OTU incidence pooling the global sample set (n = 29). Of the 317 OTUs passing filtering, 88 were present in only one sample, 64 in two samples and 34 in three samples. Distribution then reached a local maximum at six samples. Two clusters were present in all 29 samples (OTU_10, Didymellaceae, Dothideomycetes; and OTU_41, Trichomeriaceae, Eurotiomycetes, associated with DED resistance, see above), one was present in all but one (OTU_33, Cladosporiaceae, Dothideomycetes), and three others were present in all but two (**Table 1**). Beyond the category of “presence in nine samples” distribution was effectively flat. In other words, the number of OTUs present in 10 to 29 samples always ranged from 1 to 5. Note that not all samples were taken under the same conditions (single twig vs. pooled twigs).

To detect core mycobiome members, we used the independent distributions of each experiment presented in previous sections, and the incidence across all of collection sites. In that regard, 37 OTUs were found in the four sampled populations, 44 in three, 88 in two, and 153 were private to a single population. Both the pooled samples and the across-sites distributions concur with the distributions of OTUs in the clonal bank and, to a lesser extent, with that of the OTUs in the landmark tree. The OTUs present more frequently in our sampling than could be expected by chance are very likely members of the core microbiome (see Discussion). In total, 32 OTUs passed the criteria for core microbiome membership: 29 belonging to Ascomycota and three to Basidiomycota.

## 4 Discussion

### 4.1 Within-tree variation in species richness and diversity

Analyses on the landmark tree endophytic mycobiome did not reveal a clear structure, but allowed to draw some interesting conclusions: (i) although most of the samples collected displayed a similar taxonomic composition, some were remarkably different. For instance, a southern mid-height branch (H1S) was massively infected by a single OTU (**Figure 4**). (ii) The two lowest branches, resprouts from the trunk (epicormic shoots) aged a few years old, displayed higher taxonomic richness than any other branches, with a relatively higher representation of Basidiomycota. (iii) Finally, samples from the trunk showed a richness comparable to that of the crown branches. Taking this into consideration, when sampling trees to characterize their overall stem endophytic flora and to avoid considerable biases due to abnormally high local infections, we recommend pooling tissue from at least two branches. However, mixing samples from epicormic and crown branches should be avoided, because they are likely to represent different endobiome compositions. The greater richness found in the lower branches supports previous research (Andrews et al., 1980; Johnson and Whitney, 1989) and could be partly attributed to the high density of inoculum in the ground with ability of entering into the stems through roots, bark surface and stomata in leaves (Bahram et al., 2022). Similarly, as a substrate for fungi, epicormic shoots may differ in anatomy and vigor from proleptic shoots (Negrón et al., 2013).

### 4.2 Endobiome and resistance to DED

The abundance of three distinct fungal endophytic taxa was associated with higher host resistance to DED (**Table 2**). Interestingly, the two highest associations at family level (Buckleyzymaceae, in Cystobasidiomycetes; Trichomeriaceae, in Eurotiomycetes) were mostly driven by OTUs considered to be members of the core microbiome (OTU_71 and OTU_41, respectively). Moreover, a trait of two out of the three taxa (Buckleyzymaceae and Bulleraceae) is that they grow, or are able to grow, as yeasts. Yeasts have the ability to systemically colonize plants and produce phytohormones and siderophores that promote plant growth and alleviate stress (Joubert and Doty, 2018; Martínez-Arias et al., 2021c). The greater abundance of these yeasts in resistant trees could improve tree resilience to DED infection, promoting resistance mechanisms to the physiological disorders caused by the pathogen. *O. novo-ulmi* also spreads systemically through the plant’s vascular system in a yeast-like phase (Nigg et al., 2015) (blastospores), even in resistant trees (Martín et al., 2019a), inducing vessel embolism. Our results suggest that resistant trees benefit from harboring a high proportion of two fungi from the core endobiome (OTU_71 and OTU_41), which have the capacity to extensively colonize the plant. Extensive or systemic spread of an endophyte could allow higher interaction with the pathogen throughout the plant, and possibly a higher level of interaction with the plant’s physiological functions.

The first endophyte was assigned to *Buckleyzyma aurantiaca*, based on the sequence similarity to the accessions in the database UNITE. When the ITS sequence of this OTU was run against Genbank, equal hits were returned for several accessions identified as *Buckleyzyma* and *Rhodotorula*, both cultured and uncultured, but with a level of identity of 97.22% (140/144 bp). This OTU is likely to be an undescribed species. Cystobasidiomycetes is a group of basidiomycetous yeasts with unclear systematics that includes strains previously isolated from plants (Oberwinkler, 2017), soils and waters (Jones, 2011; Duarte et al., 2015; Jones et al., 2015). An elm endophytic yeast from Cystobasidiomycetes was shown to reduce *O. novo-ulmi* growth *in vitro*, partly due to the release of volatiles (Martínez-Arias et al., 2021c). Furthermore, its inoculation into elm plantlets in tandem with a Chaetothyrial yeast, favored root development, photosynthesis and survival against abiotic stress (Martínez-Arias et al., 2021b).

The second endophyte (OTU_41) was assigned to *Knufia* by our pipeline. In Genbank, it did not retrieve perfect identities, obtaining a maximum identity of 97.55% (196/201 bp) and three gaps to *Knufia* but also to genus *Exophiala*. Most accessions were derived from uncultured strains, and some from molecular studies in soils and plants. This OTU could therefore also belong to an undescribed species. The Trichomeriaceae (Chaetotyriales) were formerly part of the Herpotrichiellaceae, which have been reported to grow in the sexual phase in dead plants and wood (Geiser et al., 2006). Members of Chaetotyriales can be classified as dark septate endophytes, which can provide important benefits to their hosts as reducers of biotic or abiotic damages (Punja and Utkhede, 2003; de Tenório et al., 2019).

The third associated taxon was represented by two OTUs (OTU_70 and OTU_55) of the genus *Cryptococcus* (via BLAST to NCBI; 100% and 97% of identity, respectively) or *Genolevuria* (via dada2 to UNITE), both Tremellal yeasts frequently found in plants and water (Jones et al., 2015). Albrectsen et al. (2018) found *Cryptococcus* as an endophyte in beetle-damaged *Populus tremula* leaves. In addition, *Cryptococcus* apparently outcompetes the Rosaceae pathogen *Botrytis cinerea* due to niche occupancy (Zambell and White, 2017).

### 4.3 Phenotypic vitality and wood mycobiome

The study of the natural population with varying degrees of dieback brought out some notable taxa. Firstly, *Geosmithia* spp. was extremely abundant in the declining tree RIV2. Concurringly, it was identified as the dominant fungi in a *U. minor* tree with extensive dieback symptoms in the absence of DED pathogens (Hänzi et al., 2016). Certain *Geosmithia* fungi could therefore act as opportunistic or latent pathogens in elms, as previously reported by Hänzi et al. (2016). The presence of this genus in the healthy tree (RIV5) suggests that it is able to live as an endophyte in latent pathogenicity. Pepori et al. (2018) found that elms inoculated with *Geosmithia* fungi remained largely asymptomatic, and joint inoculation of *Geosmithia* and *O. novo-ulmi* reduced wilting symptoms compared to inoculation with *O. novo-ulmi* only. They also found parasitic behaviour of *Geosmithia* towards *O. novo-ulmi*. In elms, *Geosmithia* was frequently found in DED-infected trees (Pepori et al., 2015), most likely carried there by the beetles that are also the vectors of DED pathogens. Further research is needed into the potential contribution of *Geosmithia* to tree dieback in Rivas or, in contrast, the potential role of this taxon in the phenotypic avoidance of DED found in this elm stand.

Secondly, two other trees with dieback symptoms (RIV6 and RIV4) were dominated by Nectriaceae (especially RIV4). OTU_92 (*Fusarium*) was responsible for this signature and was also very abundant in the healthy RIV3. The family Nectriaceae (Sordariomycetes) includes facultative parasites that cause stem cankers, and saprobes. In elms, dieback symptoms have been associated with colonization by *Nectria* sp. (Heybroek, 1993; Plante and Bernier, 1997).

### 4.4 Core microbiome and among-site variation

Sampling from different spots in a single tree and from genetically different trees enabled the detection of robust signatures of a core microbiome. Out of the 231 OTUs found in the landmark tree, 11 were present in all samples (10) and 22 in more than seven samples (**Table 1**). In the clonal bank, eight OTUs were present in eight trees, seven were present in nine trees and another seven were in all trees (10). In the landmark tree and the clonal bank, the number of OTUs did not decrease following the pattern expected by randomness. The number of OTUs reached a tableau beyond five samples in both distributions (**Figures 3A,B**), and a relative maximum at the end of the distribution in the landmark tree (**Figure 3A**). Therefore, the probability that a given sample would contain a specific OTU depended on the OTU in question. Thus, not all OTUs can be considered rare events (i.e. events that would display Poisson distributions). Others with high probabilities of occurrence displayed different distributions (Poisson distributions, but with “absence of OTU” as rare event). Although not appreciable, perhaps due to their low numbers, other OTUs may have behaved as “medium frequency events”, retrieving binomial distributions. Thus, the lack of agreement between the observed distributions and the expected monotonic decrease, characteristic of pure Poisson processes, shows that OTU occurrences range from rare to highly frequent. OTUs that follow a pattern of occurrence consistent with a Poisson distribution could be considered local infections with arguably different but low likelihoods of infecting a stem. Highly frequent OTUs, on the other hand, are likely to be members of the core microbiome. It is unclear why this latter group of endophytes is pervasive, but it could be explained by a high infective capacity (Griffin and Carson, 2018) (e.g. through insect vectors, rain and wind) and/or systemic propagation within the plant, as occurs in some endophytic yeasts (Joubert and Doty, 2018). Shallower sampling may not have allowed us to distinguish between the two trends in OTU occurrence, because the distributions would have overlapped, obscuring the underlying pattern. The most commonly found fungal taxa both in the landmark tree and the clonal bank were the ascomycetous classes Dothideomycetes, Eurotiomycetes, Sordariomycetes, Leotiomycetes and Lecanoromycetes, and the basidiomycetous classes Tremellomycetes and Cystobasidiomycetes.

We identified 32 core OTUs by defining the core microbiome as the OTUs that are present in at least eight out of 10 samples in either the landmark tree or the clonal bank, and present in at least two populations. Although most of them were present in most samples across the four populations, some were abundant in the clonal bank but rare or absent in the landmark tree (e.g. OTU_18 and OTU_38). Considering that the clonal bank includes trees from various provenances across Spain (**Supplementary Table 1**) and a few are from the same provenance as the landmark tree, it is conceivable that these OTUs are controlled mostly by environmental cues (Zimmerman and Vitousek, 2012). Conversely, a few OTUs were widespread in the landmark tree, but rarer in the clonal bank (e.g. OTU_66, OTU_80 and OTU_102). OTU_66 and OTU_80 were present in the four populations and most of the samples but surprisingly lacking in some trees from the clonal bank. This pattern hints at an implication of host genotype (see Bálint et al. (2013)). However, physiological status and microscale environmental variation could also explain this pattern. The clear separation of samples by site shown in the Principal Component Analysis (**Figure 2**) indicates the important role of geographical location in shaping fungal endobiome communities. New targeted experiments are needed to confirm or refute these hypotheses.

## 5 Concluding remarks

We found clear evidence of the existence of a core endophytic mycobiome in elm stems, which account for circa 10% of the total endophyte richness. Our study strongly suggests that some core endophytes are associated to DED resistant genotypes. Recent works have shown the beneficial role of some endophytic yeasts in *U. minor* resilience against stress and in priming defenses against *O. novo-ulmi* (Martínez-Arias et al., 2021a). Therefore, resistant trees could not only display inherent genetic mechanisms of resistance, such as narrow earlywood vessels (Martín et al., 2021) or an early molecular response against the pathogen (Sherif et al., 2016), but could also benefit from mechanisms of resistance provided by their symbiotic microbiome. If this microbiome were heritable, new possibilities for elm breeding could arise directed to improve microbial functioning. Otherwise, the possibility of transplanting beneficial microbiomes could open new prospects for the fight against the disease.

## Data availability statement

The datasets of the demultiplexed raw reads for this study can be found in the European Nucleotide Archive with the accession number PRJEB58145. R scripts used for this study and some processed datasets are stored at https://github.com/dmacaya/core-elm-mycobiome.

## Author contributions

DM-S and JM contributed to the conception and design of the study. DM-S and JM performed the sampling and sample processing. CC supervised the molecular work at the lab. DM-S performed the molecular work, bioinformatics and statistical analysis. DM-S and JM wrote the draft of the manuscript. JW, CC, and LG revised the manuscript. All authors contributed to the article and approved the submitted version.
