# Supplementary material for: Core endophytic mycobiome in Ulmus minor and its relation to Dutch elm disease resistance
Source: Front Plant Sci. 2023 Feb 28;14:1125942. doi: 10.3389/fpls.2023.1125942 (PMC10011445; doi:10.3389/fpls.2023.1125942)
Supplement: Supplementary file 1 [file DataSheet_1.docx]

**Supplementary Information**

Sup. Fig. 1. Pictures of trees from the Rivas natural population.

Sup. Fig. 2. Rarefaction curves for the 29 samples collected.

Sup. Fig. 3. Rarefaction curves grouped by collection site.

Sup. Table 1. Susceptibility to DED in the trees of the clonal bank.

Sup. Text. Detailed methods.

Supplementary references.

1. RIV1


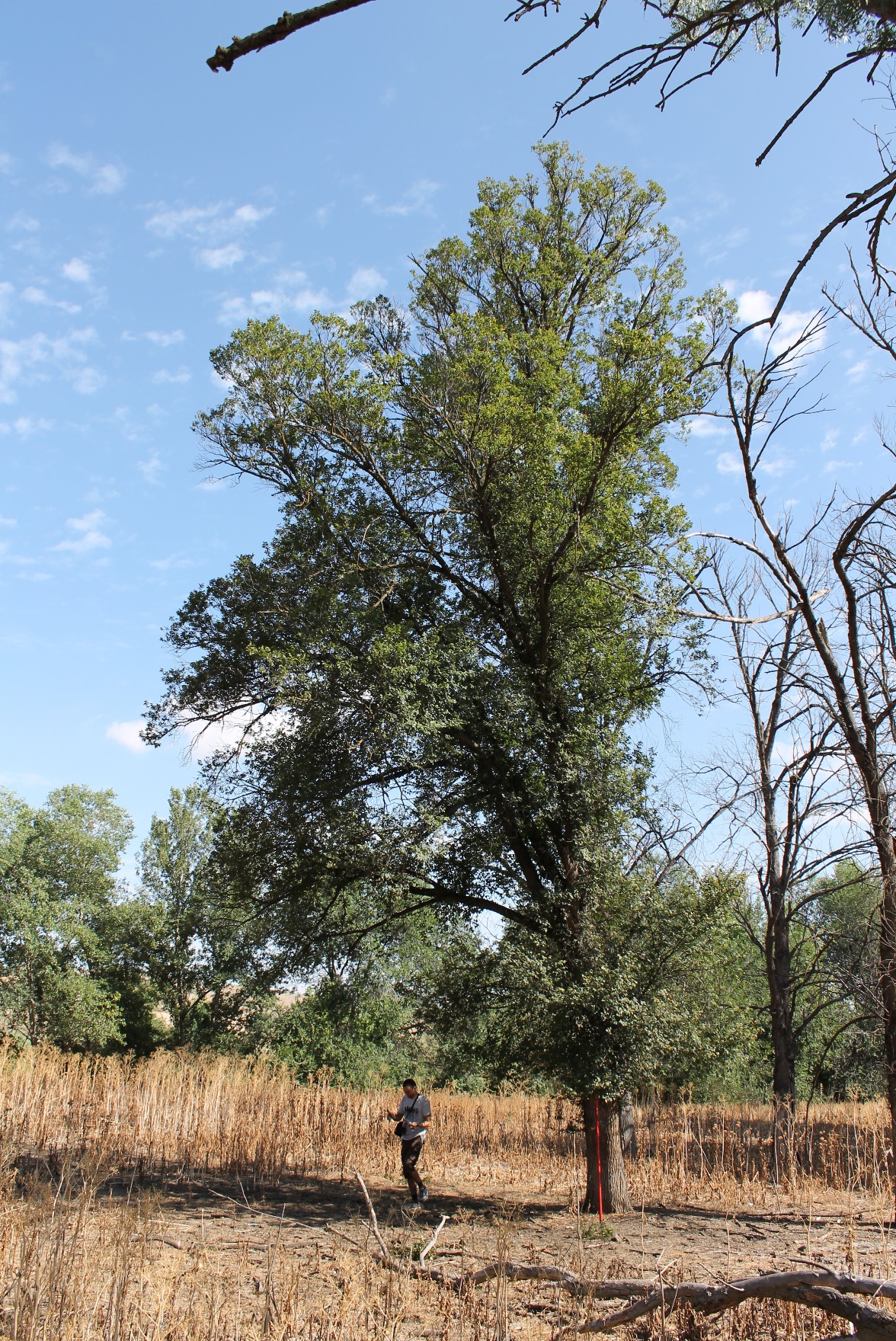


1. RIV2


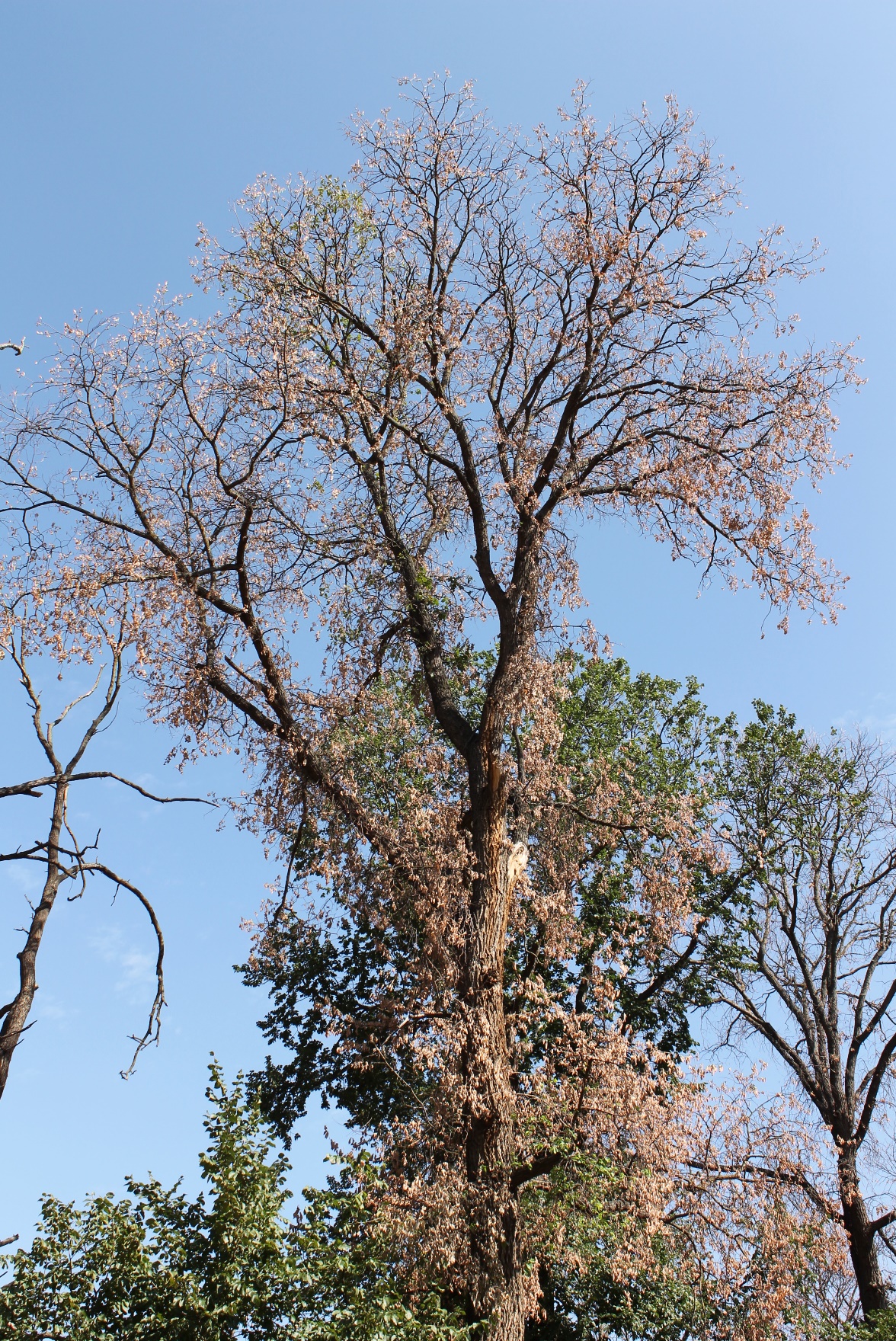


1. RIV3


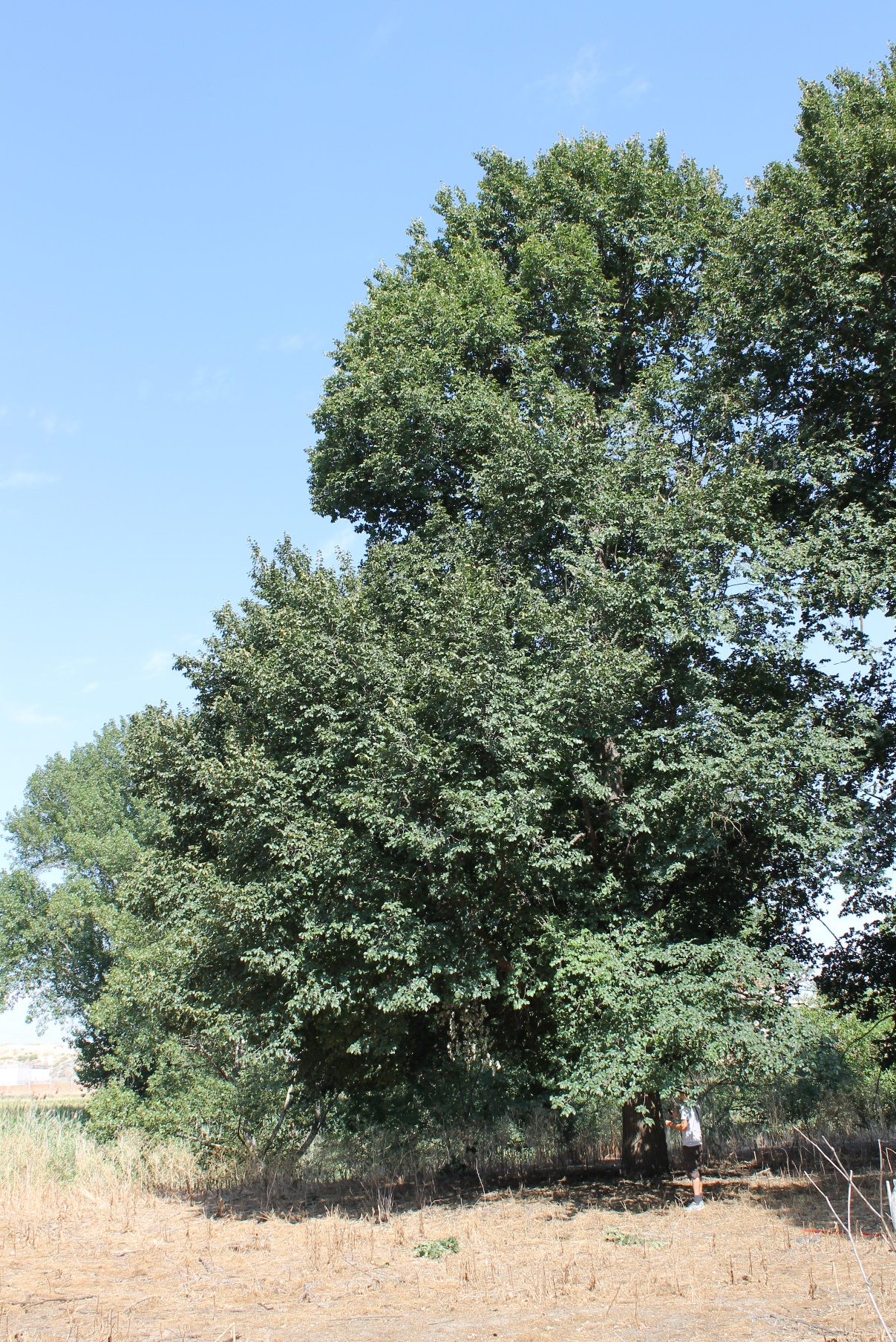


1. RIV4


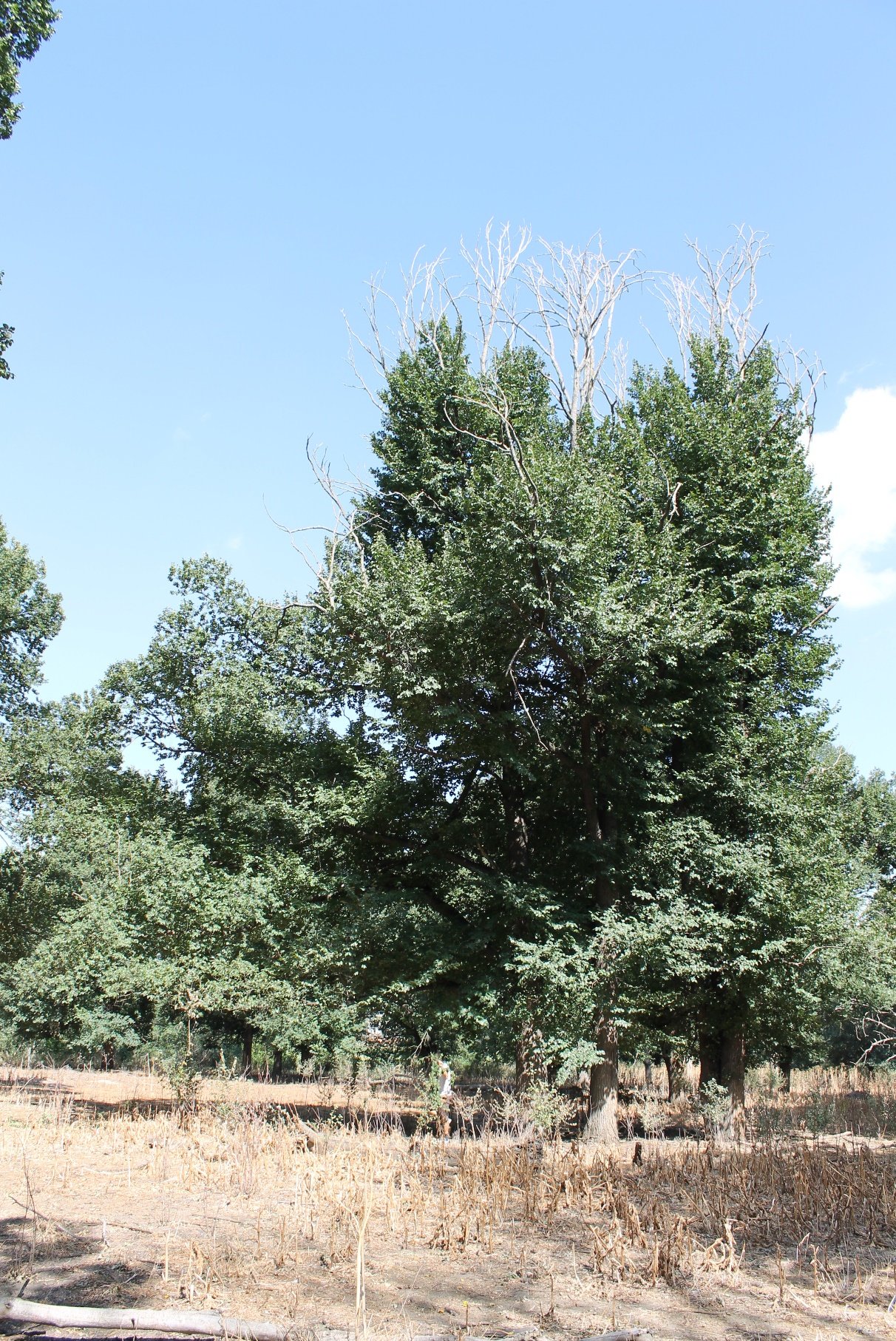


1. RIV5


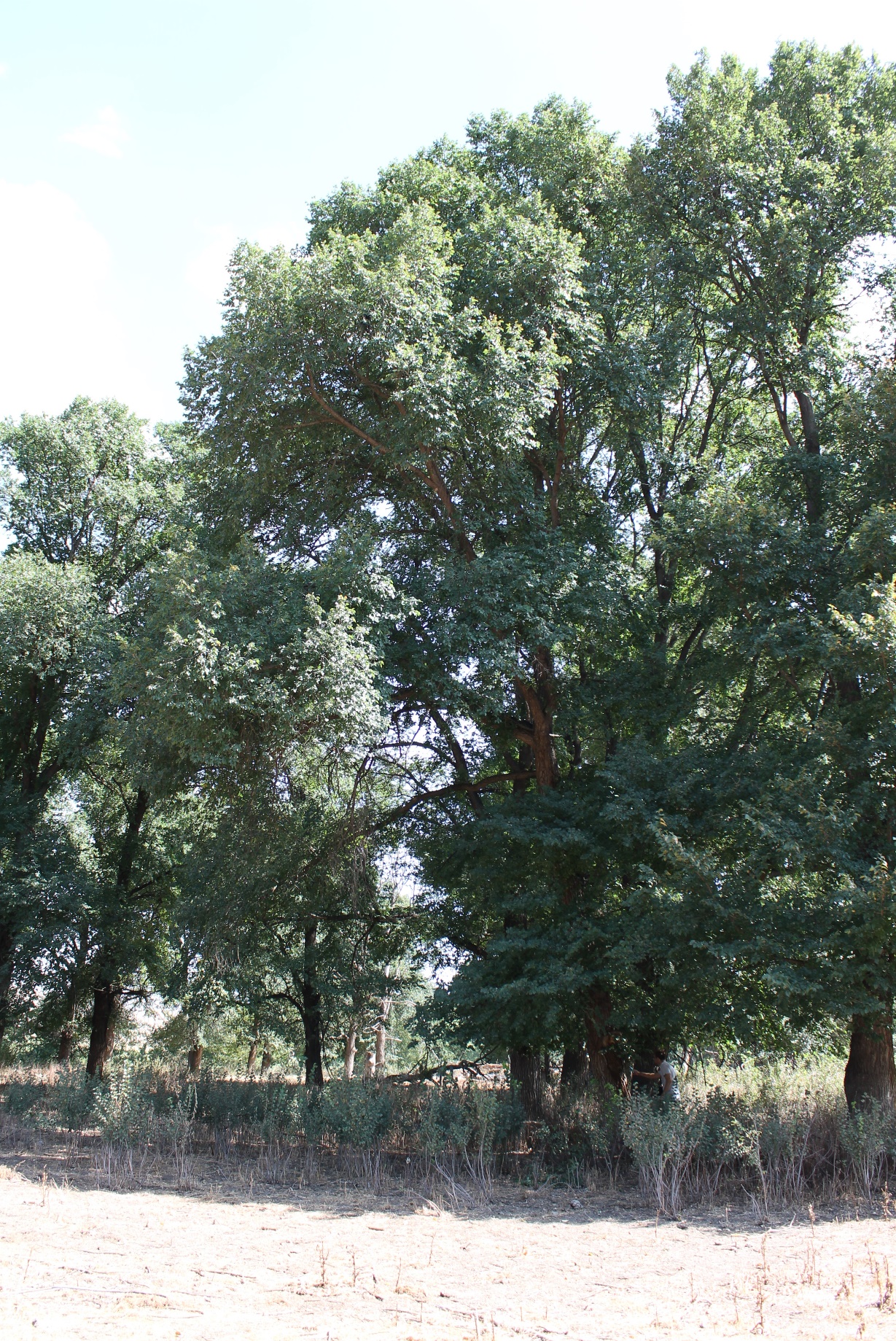


1. RIV6


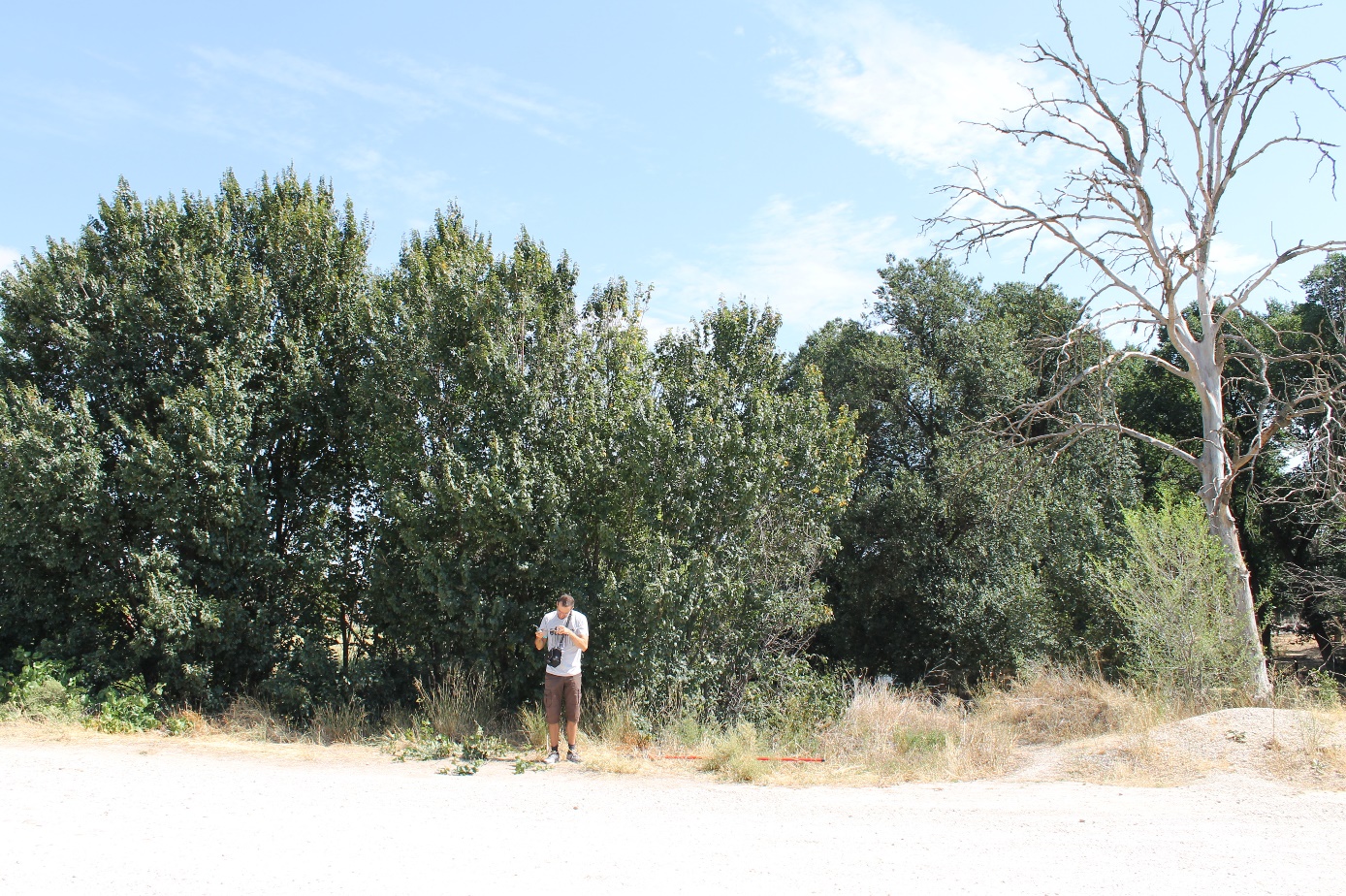


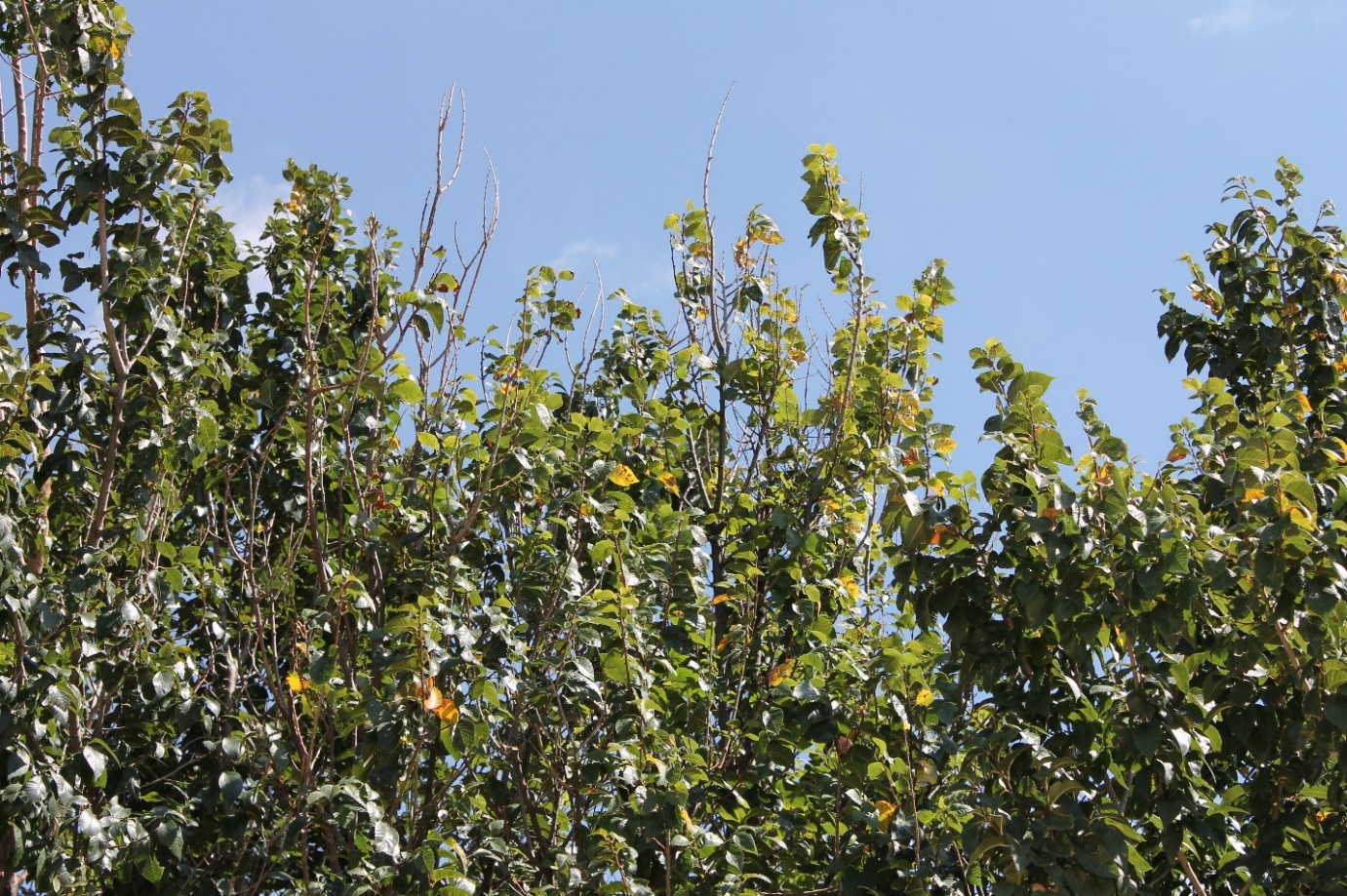


**Sup. Fig. 1.** Trees from the Rivas natural population during sample collection: A) RIV1; B) RIV2; C) RIV3; D) RIV4; E) RIV5; F) RIV6.


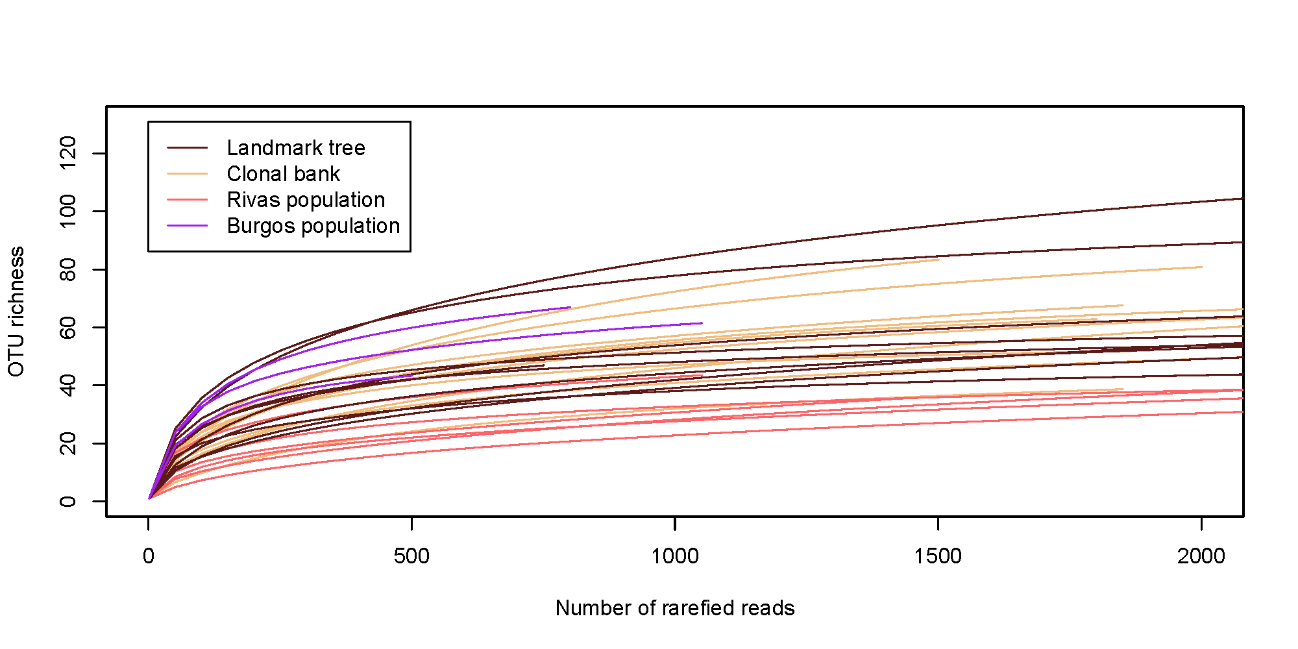


**Sup. Fig. 2.** Rarefaction curves for the 29 samples collected, displaying an increasing sequencing effort from 0 to 2000 reads.


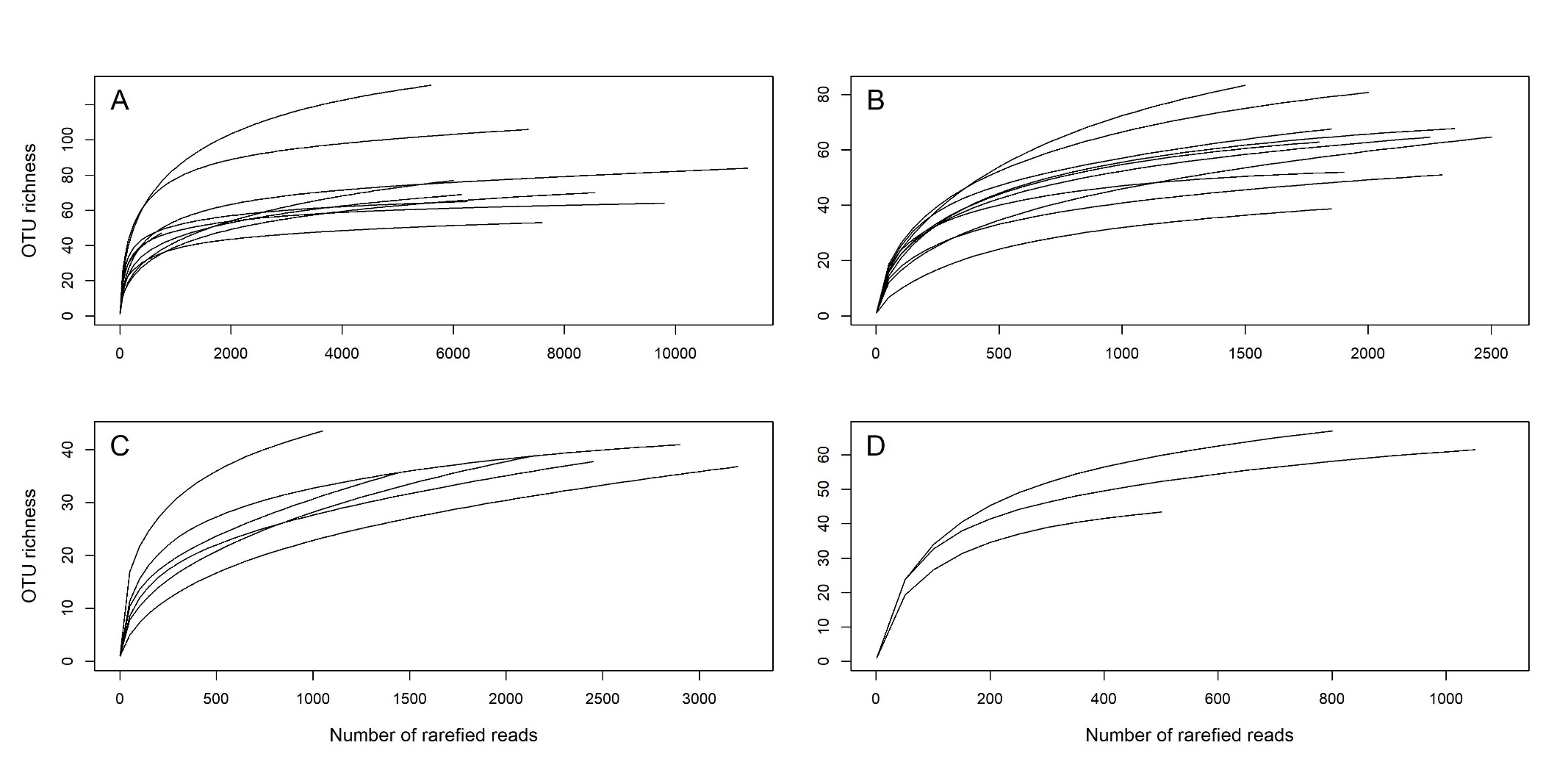


**Sup. Fig. 3.** Rarefaction curves for the samples collected, grouped by collection site, showing an increasing sequencing effort. A) Landmark tree; B) Clonal bank; C) Rivas population; D) Burgos population.

**Sup. Table 1.** Tree resistance to DED, measured as the percentage of the foliage wilting, shown by the 10 selected *Ulmus minor* genotypes in different inoculation tests performed at Puerta de Hierro Forest Breeding Centre (Madrid, Spain). In each year, foliage wilting values were recorded 60 days after inoculation with *Ophiostoma novo-ulmi*.

| **Genotype** | **Origin in Spain** | **Foliage wilting percentage (mean ± SE) (and number of replicates)** | | | | | | **Average foliage wilting (%)** |
| --- | --- | --- | --- | --- | --- | --- | --- | --- |
|  |  | **Year 2002** | **Year 2008** | **Year 2009** | **Year 2010** | **Year 2011** | **Year 2012** |  |
| CR-RD2 | Ruidera, Ciudad Real | 93.7 ± 8.3 (8) | - | - | - | - | - | 93.7 ± 8.3 |
| GR-DF3 | Deifontes, Granada | 37.6 ±8.9 (7) | - | 28.7 ± 4.8 (9) | 47.7 ± 7.4 (9) | 34.4 ± 4.4 (9) | - | 37.1 ± 4.0 |
| GR-HL2 | Huélago, Granada | 81.2 ± 8.3 (8) | - | - | - | - | - | 81.2 ± 8.3 |
| J-CA2 | Jaen, Cazorla | 30.0 ± 7.4 (8) | - | 29.0 ± 5.5 (10) | 40.5 ± 6.7 (10) | - | - | 33.2 ± 3.7 |
| MA-PD2 | Málaga, Pedrizas | 36.2 ± 8.3 (8) | - | - | - | 43.5 ± 4.1 (10) | 49.5 ± 7.8 (10) | 43.1 ± 3.8 |
| M-DV1 | Dehesa de la Villa, Madrid | 82.5 ± 8.4 (7) | 89.2 ± 6.4 (10) | 87.1 ± 5.4 (10) | - | 86.7 ± 4.9 (8) | 80.0 ± 6.3 (8) | 85.1 ± 1.7 |
| M-DV5 | Dehesa de la Villa, Madrid | 28.1 ± 8.3 (8) | - | 12.5 ± 5.6 (10) | 13.5 ± 6.9 (10) | - | - | 18.0 ± 5.0 |
| M-RT1.5 | Retiro, Madrid | - | 13.2 ± 2.6 (3) | 19.8 ± 7.9 (3) | - | 14.0 ± 5.2 (7) | 14.2 ± 5.8 (7) | 15.3 ± 1.5 |
| TO-PB1 | Toledo, Puebla de Montalbán | - | - | - | - | 64.5 ± 5.4 (6) | 100.0 ± 0.0 (6) | 82.2 ± 17.8 |
| V-AD2 | Ademuz, Valencia | - | 10.9 ± 7.0 (10) | 17.2 ± 4.6 (10) | - | - | - | 14.0 ± 3.2 |

**Sup. Text.** Detailed methods

### Level of resistance of Ulmus minor genotypes

Several replicates of each clone (*N* = 3-10; Sup. Table 1), previously planted in several field experimental plots at Puerta de Hierro Forest Breeding Centre (Madrid), were artificially inoculated with an aqueous suspension of *O. novo-ulmi* blastospores, following the inoculation methodology described in Martin et al. (2015). In brief, mycelial plugs from fresh colonies of the pathogen were grown in Erlenmeyer flasks with Tchernoff’s liquid medium (Tchernoff, 1965) under constant shaking at 22 ^o^C to induce sporulation. The spores were collected by centrifugation and resuspended in sterile distilled water. The concentration was adjusted to 10^6^ blastospores ml^-1^. Two drops of the inoculum were delivered into the xylem of each tree through a transverse cut made with a sharp blade at 5 cm from the substrate level, allowing the absorption of the inoculum. To perform the inoculations, fresh local cultures of the pathogen were annually obtained during late winter or early spring from xylem of elms that were heavily diseased in the previous season. All screening tests were performed with trees of at least four years of age and in early spring (25 April – 10 May), coinciding with peak susceptibility of elms in Madrid (Martín et al. 2021). The percentage of foliage wilting in the crown was recorded at day 60 after *O. novo-ulmi* inoculation by three independent observers. The average value of foliage wilting was used as a proxy to the resistance to DED of each clone. None of the trees examined for endophyte composition were inoculated with the pathogen.

### DNA isolation

All the samples were stored below 10°C and processed in a laminar flow chamber within the next 24 hours. First, they were surface-sterilized, with sequential baths: (1) ten minutes lightly shaken in autoclaved distilled water with a drop of Tween 20, (2) once dried, 30 seconds in ethanol 75%, (3) five minutes in sodium hypochlorite 4%, and (4) 15 seconds in ethanol 75% and left to dry. Afterwards, they were carefully peeled with a sterilized scalpel, removing the periderm. The wood samples were wrapped in autoclaved aluminum foil and stored at –80°C. Later, the samples were ground with an IKA A11 mill (Staufen, Germany), keeping them frozen with the regular addition of liquid nitrogen. The wood powder was stored at –80°C in sterile centrifuge tubes. The two wood cores from the trunk of the landmark tree had the rhytidome removed. Later they were ground with autoclaved pestles and mortars with regular addition of liquid nitrogen to keep the tissue continuously frozen.

DNA isolation was done after wood powder enzymatic digestion to improve recovery of fungal DNA. Approximately 150 mg of wood powder was incubated for 20 minutes at 37°C in 400 µl of enzymatic solution containing 0.04 mg of Lyticase (ref. n. L2524; Sigma-Aldrich, St. Louis MO, USA) and Tris-EDTA 50 mM. Afterwards, the samples were centrifuged at 13,000 rpm and the supernatant removed. The rest of the DNA isolation was carried out using the Invisorb Spin Plant Mini Kit (STRATEC Biomedical AG, Birkenfeld, Germany) following manufacturer instructions, with the following modifications: after Lysis Buffer, a pinch of zirconium oxide beads 0.1 mm diameter (ref. n. 1005-02; Bertin Instruments, France) was added, and during the subsequent 65°C bath the samples were vortexed three times. These modifications were done to increase fungal cell wall disruption, therefore improving the recovery of fungal DNA.

### DNA amplification

The profiling of the endophyte composition was carried by high throughput sequencing of the first internal transcribed spacer region (ITS1) of the ribosomal DNA. The amplification was done in two steps. First, we amplified the target region with oligonucleotides that contained the specific fungal primer ITS1-F (Gardes and Bruns 1993) or the non-specific primer ITS2 (White et al. 1990) on the 3’ edge and universal tails on the 5’ edge. Afterwards, the amplified product was re-amplified with oligonucleotides containing such universal tails, a sample-specific tag and the adaptors for 454-pyrosequencing chemistry. Given that amplifications of the two main sample sets (monumental tree and clonal bank) were done at different times, we were forced to use different polymerases (TaKaRa PrimeSTAR GXL and Roche FastStart High Fidelity, respectively), so the protocols varied slightly. This could rise some concerning in terms of comparative power. However, since this study just pretended to contrast these two datasets primarily in terms of incidence, and not of abundance, different PCR chemistries do not pose a problem. The populations of Rivas-Vaciamadrid and Burgos were assayed with the same conditions as the ones of the clonal bank. Such conditions for the first and the second PCR respectively were: a starting denaturation at 95°C for 2 min; then 35 cycles of 95°C for 30 s, 55°C for 30 s and 72°C for 1 min; finishing with a final elongation step of 72°C for 5 min. For both PCRs, the reactions were done for a volume of 15 µL with final concentrations of ~5ng/µL of total isolated DNA or ~0.1ng/µL of product from the first PCR (1 µL of stock DNA solution or 1 µL of product of the first PCR diluted 1/100), 1× of reaction buffer, 1.8 mM of MgCl_2_, 0.2 mM of each dNTP, 0.4 mM of each primer and 0.05 U/µL of FastStart enzyme. The conditions for the PrimeSTAR polymerase were as specified in the manufacturer manual for the 3-step PCR, setting Tm to 58°C in the first PCR and 60°C in the second, and increasing the cycles to 35 in the first PCR. Reaction volume was 15 µL.

### High-thoughput sequencing

We decided the survey on the landmark tree to be the first one because depicting the endophytic mycobiome within a tree would provide information about the sampling effort needed in subsequent experiments to properly characterize each tree mycobiome. Since we did not have a priori information about how deep we should sample, and we considered that deciphering the within-tree endophytic structure deserved a higher resolution, we allotted a large sequencing capacity to this purpose. Given that in the clonal bank experiment we aimed at detecting possible associations to DED tolerance not only of the abundant endophytes but also of the rare ones, such experiment also received an important sequencing allocation to attain a considerable resolution. The elm population of Burgos, on the other hand, was shallowly sequenced, because it was used mostly as an outgroup, to reveal how ubiquitous the core microbiome detected in the other three populations was. After the second PCR, the product of all the samples was quantified, pooled equimolarly and pyrosequenced in a 454 GS FLX Titanium platform (Roche, Switzerland, Basel). The sequencing effort was as follows: monumental tree samples were run in 1/8^th^ of a plate, clonal bank ones in the 62.5% of 1/8^th^ of a plate, Rivas-Vaciamandrid 37.5% of 1/8^th^ of a plate and the Burgos population was run in the 4% of 1/4^th^ plate.

### Default values for RunTitanium script from AmpliconNoise v1.29 (Quince et al. 2011)

Parameters spyro=60 and cpyro=0.01 for PyroNoise; sseq=25 and cseq=0.08 for SeqNoise; and alpha=-7.5 and beta=0.5 for Perseus. The minimum and maximum size of flowgram sizes were 360 and 720 bp, respectively. Sequences were truncated to 400 bp.

### Visualization of data and test of hypothesis with DeSeq2

To visually explore for general patterns and groupings, we used the multivariate methods included in the package. First, we apply to the count data a Variance Stabilizing Transformation. Later, the adjusted counts were used to reduce the dimensionality of the dataset through a Principal Component Analysis and to cluster the samples. Afterwards, we test the hypothesis whether any of the taxonomic groups was associated with the tolerance to DED. We implemented the model in DESeq2 setting wilting (a proxy of DED tolerance) as the only explanatory variable. These tests were conducted at OTU, family, order and class levels, and only for groups with more than 5 counts adding up the ten clonal bank samples.

At the OTU level, we deemed as significant but to a lower degree passing the threshold of FDR < 0.15: though being significant in the independent tests, the significance was weaker when accounting for multiple-testing. We consider those reports with recording.

**Supplementary references**

Gardes M, Bruns TD (1993) ITS primers with enhanced specificity for basidiomycetes - application to the identification of mycorrhizae and rusts. Mol Ecol 2:113–118. doi: 10.1111/j.1365-294X.1993.tb00005.x

Martin JA, Solla A, Venturas M, et al (2015) Seven *Ulmus minor* clones tolerant to *Ophiostoma novo-ulmi* registered as forest reproductive material in Spain. iForest - Biogeosciences For 8:172–180. doi: 10.3832/ifor1224-008

Quince C, Lanzen A, Davenport RJ, Turnbaugh PJ (2011) Removing Noise From Pyrosequenced Amplicons. BMC Bioinformatics 12:38. doi: 10.1186/1471-2105-12-38

Tchernoff V (1965) Methods for screening and for the rapid selection of elms for resistance to Dutch elm disease. Acta Botanica Neerlandica 14:409–452. https://doi.org/10.1111/j.1438-8677.1965.tb00204.x

White TJ, Bruns T, Lee S, Taylor J (1990) Amplification and direct sequencing of fungal ribosomal RNA genes for phylogenetics. In: Innis MA, Gelfand DH, Sninsky JJ, White TJ (eds) PCR protocols: a guide to methods and applications. Academic Press, San Diego, pp 315–322
